# ﻿Revision of *Trigastrotheca* Cameron (Hymenoptera, Braconidae, Braconinae) with descriptions of 13 new species

**DOI:** 10.3897/zookeys.1205.125014

**Published:** 2024-06-21

**Authors:** Donald L. J. Quicke, Simon Van Noort, Avunjikkattu Parambil Ranjith, Ariel L. L. Friedman, Hans Mejlon, Buntika A. Butcher

**Affiliations:** 1 Integrative Insect Ecology Research Unit, Department of Biology, Faculty of Science, Chulalongkorn University, Pathumwan, Thailand Chulalongkorn University Pathumwan Thailand; 2 Research and Exhibitions Department, South African Museum, Iziko Museums of South Africa, P.O. Box 61, Cape Town, 8000, South Africa South African Museum, Iziko Museums of South Africa Cape Town South Africa; 3 Department of Biological Sciences, University of Cape Town, Private Bag, Rondebosch, 7701, South Africa University of Cape Town Rondebosch South Africa; 4 The Steinhardt Museum of Natural History, Tel Aviv University, Tel Aviv 69978, Israel Tel Aviv University Tel Aviv Israel; 5 Museum of Evolution, Zoology, Uppsala University, Norbyvägen 16, SE-752 36 Uppsala, Sweden Uppsala University Norbyvägen Sweden

**Keywords:** Identification key, molecular phylogeny, morphology, parasitoid, taxonomy

## Abstract

The Old World braconine wasp genus *Trigastrotheca* Cameron is revised. The genus is recorded from the island of Madagascar for the first time based on two new species, *T.christianhenrichi* Quicke & Butcher, **sp. nov.** and *T.formosa* Quicke & Friedman, **sp. nov.***Trigastrothecagriffini* Quicke, **sp. nov.** is described from Australia; *T.aethiopica* Quicke & Friedman, **sp. nov.** is described from Ethiopia; *T.braeti* Quicke & Butcher, **sp. nov.** is described from Congo; *T.simba* van Noort, **sp. nov.** is described from Tanzania; *T.freidbergi* Quicke & Friedman, **sp. nov.**, *T.carinata* Ranjith, **sp. nov.**, *T.flava* Ranjith, **sp. nov.** and *T.similidentata* Ranjith, **sp. nov.** are described from India; *T.khaoyaiensis* Quicke & Butcher, **sp. nov.**, *T.naniensis* Quicke & Butcher, **sp. nov.**, and *T.sublobata* Quicke & Butcher, **sp. nov.** are described from Thailand. *Trigastrothecatridentata* is recorded from Thailand for the first time. A putative female of *T.sureeratae* is described for the first time. *Acroceriliatricolor* Quicke & Ingram, 1993 is transferred into *Trigastrotheca*, as *T.acroceropsis***nom. nov.** A key is provided for the identification of species.

## ﻿Introduction

*Trigastrotheca* Cameron is a small genus of highly distinctive parasitoid wasps known mainly from the Indo-Australian region (Enderlein 1920; Quicke et al. 2017; Raweearamwong et al. 2020) but also occurring in Africa and Australia (Quicke and Ingram 1993). The unique morphological characteristic of *Trigastrotheca* is the modified posterior margin of the fifth metasomal tergite with strong submedial posterior emarginations giving rise to a medial and pair of sublateral points in the female (Quicke 1987). The otherwise similar males lack these modifications and have rather long and posteriorly weakly convex T5. The latter led van Achterberg (1983) to describe a male of a species from west Africa (Sierra Leone) in a separate genus, *Kenema* van Achterberg. Quicke (1987), having seen series comprising both sexes, considered *Kenema* to be a junior subjective synonym of *Trigastrotheca*. However, van Achterberg and Sigwalt (1987) did not follow this and described a second species under the generic name *Kenema*, from Senegal based only on a male and provided a key to separate the two genera. We reject this, as did Samartsev (2023) as the characters employed are either sexually dimorphic or weak, and further, the two species placed in *Kenema* both being from West Africa, could well just display characters of a local species complex.

Until recently, the only species described from tropical Asia was *T.tridentata* (Enderlein, 1920) from Indonesia (Sumatra), the type species of Enderlein’s genus *Odontopygia* Enderlein, 1920. *Odontopygia* was synonymised with *Trigastrotheca* by Quicke (1987), although the combination of *T.tridentata* was not actually published until Quicke and van Achterberg (1990). Quicke et al. (2017) considered a specimen of *T.tridentata*, from India to represent a range extension; however, careful re-examination has revealed several further differences and we herein describe it as another new species. *Trigastrothecacostator* Thunberg, 1822, from South Africa, was originally described in the genus *Ichneumon* and later made the type species of *Coelodontus* Roman, 1912, and this was also synonymised with *Trigastrotheca* by Quicke (1987). *Trigastrothecacostator* was later renamed *T.romani* Quicke, 2005, due to homonymy within the genus *Ichneumon* (Quicke and Stanton 2005).

Raweearamwong et al. (2020) provided a checklist to the 15 species recognised at the time, which included eight from the Afrotropical region. Of these *Mesobraconinermis* (Guérin-Meneville, 1848), originally described in the Rogadinae genus *Spinaria*, was transferred to *Trigastrotheca* by Quicke and Stanton (2005) based on the short original description which mentions a dentate posterior metasomal margin of T5 but which lacked illustrations. The species was subsequently listed as such by Yu et al. (2016) and by Raweearamwong et al. (2020). One of us (DLJQ) had overlooked that in his revision of Afrotropical rogadine braconid genera, van Achterberg (1991) who had examined the type specimen, which is deposited in Zoologische Staatssammlung München, Germany and transferred *S.inermis* to the braconine genus *Mesobracon* Szépligeti. Therefore, we do not consider this species further here.

Quicke and Koch (1990) transferred *Habrobraconrugosus* Szépligeti, 1914, from Tanzania to *Kenema*. and to *Trigastrotheca* (Quicke and Stanton 2005), and recently, Samartsev (2023) transferred *Habrobraconnotata* Szépligeti, 1914, from Equatorial Guinea, to *Trigastrotheca*. Both species, are based only on their male holotypes in the MNB, and are therefore excluded from the present study.

The only known host record is for the East African *T.laikipiensis* Quicke which is an idiobiont brood parasite attacking eggs, larvae, and pupae of acacia-ants, predominantly *Crematogaster* Lund, 1831, species (Quicke and Stanton 2005; Stanton et al. 2005). However, since acacia ants do not occur in Asia or Australia, their hosts there must belong to other groups. One possibility might be ant plants such as *Myrmecodia* species (Rubiaceae).

Here we consider the world fauna as a whole and recognise a total of 26 described and valid species of which we describe and illustrate 13 as new and transfer one species from *Acrocerilia* van Achterberg, 1989, to the genus. We present a key to the world species although excluding Afrotropical members known only from males which were described under the generic name *Kenema* since we are currently unable confidently to integrate into the taxonomy either by associating with similarly colored species described from females or confirming that they are indeed separate species. Additionally, notes are provided on *T.acroceropsis* nom. nov., *T.trilobata*, and *T.romani*, and their holotypes illustrated photographically. The female specimen of *T.sureeratae* is described for the first time.

## ﻿Materials and methods

Terminology follows van Achterberg (1988) except for wing venation nomenclature which follows Sharkey and Wharton (1997); see also fig. 2.2 in Quicke (2015) for comparison of wing venation naming systems. Femur lengths were measured excluding the trochantellus. Metasomal tergite/tergites are abbreviated as **T/TT**.

Specimens were imaged by a variety of different systems. Those of *T.christianhenrichi* sp. nov. and *T.aethiopica* sp. nov. were taken with a Leica DFC295 digital camera mounted on a Leica M205C microscope; image stacks were processed with Leica Application Suite 4.2.0 and Helicon Focus 5.3. The holotype of *T.trilobata* and types of *T.simba* sp. nov. were imaged at SAMC with a Leica LAS 4.9 imaging system, comprising a Leica® Z16 microscope with a Leica DFC450 Camera and 0.63 × video objective attached. The imaging process, using an automated Z-stepper, was managed using the Leica Application Suite V 4.9 software installed on a desktop computer. Diffused lighting was achieved using a Leica LED 5000 Dome. Specimens of *T.griffini* sp. nov. were imaged using a Keyence VHX-7000 Digital microscope and Keyence image stitching software. Images of *T.carinata* sp. nov., *T.flava* sp. nov. and *T.similidentata* sp. nov.were taken with Keyence VHX-6000 digital microscope. The specimen of *T.sublobata* sp. nov. was imaged using an Olympus SXZ16 microscope with an Olympus DP72 camera and images combined using the Cell^D image processing system.

Collections holding specimens are abbreviated as follows:

**AIMB** ATREE Insect Museum, Bengaluru, India;

**CUMZ**Insect Museum, Chulalongkorn University Museum of Natural History, Bangkok, Thailand;

**CNCO** Canadian National Collection of Insects, Ottawa;

**DZUC**Department of Zoology, University of Calicut, Kerala, India;

**NHMUK**Natural History Museum, London, U.K.;

**HNHM**Hungarian Natural History Museum, Budapest, Hungary;

**SMNHTAU**The Steinhardt Museum of Natural History, Tel Aviv University, Tel Aviv, Israel;

**MNB** Museum für Naturkunde, Humboldt Universität, Berlin, Germany;

**SAMC**Iziko South African Museum, Cape Town, South Africa.

### ﻿Molecular methods

Sequences for the barcoding region of cytochrome oxidase *c* subunit 1 (COI) and for the D2-D3 expansion region of 28S rDNA (28S) were generated from wasp legs by the Centre for Biodiversity Genomics, University of Guelph, based on standard protocols as described in Hebert et al. (2003), Park et al. (2010), and Quicke et al. (2023a) respectively. Alignment of COI was trivial as there were no indels. The length-variable 28S sequences were aligned according to the secondary structure model of Gillespie et al. (2005) as in other studies (Butcher et al. 2014; Quicke et al. 2016). The concatenated data set was analysed using the maximum likelihood programme RAxML-NG (Kozlov et al. 2019) with full bootstrap (100 replicates). Outgroups were selected from the clade that was recovered as immediate sister group to *Trigastrotheca* in the four gene maximum likelihood (IQ-TREE) analysis of the Braconinae by Quicke et al. (2023a), together with a representative of *Physaraia* Shenefelt, from the next closest clade.

Gene sequences are deposited in GenBank and accession numbers are given in Table 1.

**Table 1.** *Trigastrotheca* specimens used for molecular analyses with their provenances and GenBank accession numbers for sequence analysed.

Taxon	Provenance	Sample Process ID	GenBank Accessions Nos.	
			COI	28S
*T.braeti* sp. nov.	Republic of Congo	BBTH4962-22	PP782008	—
*T.doiphukhaensis*	Thailand	BBTH1811-19	ON325092	OQ848751
*T.doiphukhaensis*	Thailand	BBTH3135-22	PP782009	—
*T.formosa* sp. nov.	Madagascar	BBTH744-17	PP782007	—
*T.formosa* sp. nov.	Madagascar	BBTH743-17	MH260662	MH234981
*T.griffini* sp. nov.	Australia	NSWHP2575-19	OQ928290	OQ924399
*T.khaoyaiensis* sp. nov.	Thailand	BBTH3133-22	PP782011	—
*T.khaoyaiensis* sp. nov.	Thailand	BBTH3134-22	OQ928234	—
*T.laikipiensis*	Kenya	BBTH1634-18	ON324918	ON128915
*T.sureeratae*	Thailand	BBTH569-16	MH260659	MH234978
*T.sureeratae*	Thailand	BBTH714-17	PP782010	PP782168
*T.sureeratae*	Thailand	BBTH3136-22	PP782012	—
*T.tridentata*	Thailand	BBTH2668-21	ON324922	ON128916
*T.tridentata*	Thailand	ASQSQ481-09	HM435195	ON128916
*T.aethiopica* sp. nov.	Ethiopia	BBTH740-17	MH260693	MH235015
*Philomacroploea* sp.	Thailand	BBTH2780-21	MH260667, ON325042	MH234988
*Crinibraconchromusae*	India	DQHYM079-17	MH260687	MH235009
*Physaraia* sp.	South Africa	ETKII915-12	ON324968	ON128926
*Simplicibracon* sp.	South Africa	GMSAQ502-13	OQ928236	OQ922158
*Testudobraconlongicaudis*	Japan	GBAH22892-19	LC020125	—

## ﻿Results

### ﻿Molecular analysis

**Figure 1.**
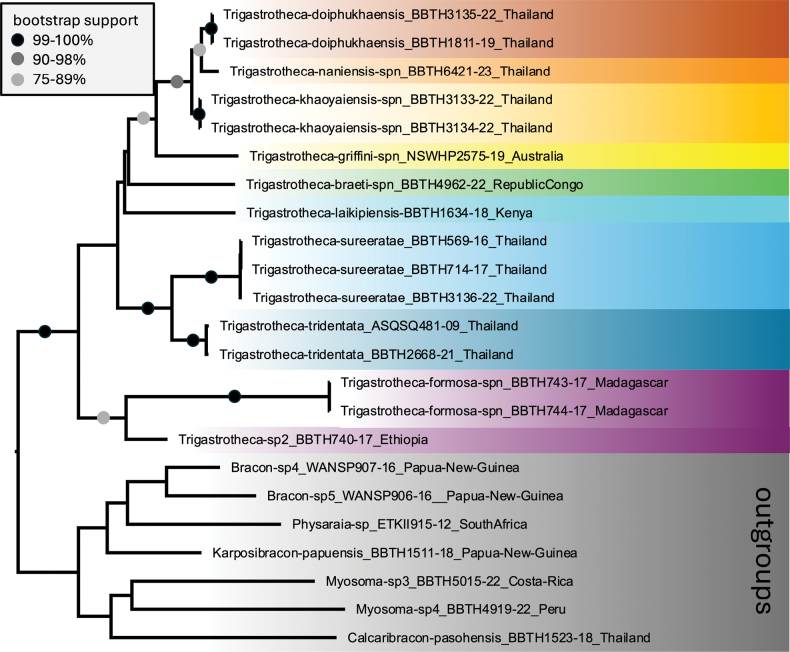
Maximum likelihood bootstrap tree of all available *Trigastrotheca* sequences rooted with a representative of the putatively closely related genera, based on analysis of concatenated cytochrome oxidase (COI) and 28S rDNA sequences.

The most likely phylogenetic tree obtained by analysis of the concatenated data set is shown in Fig. 1 with bootstrap support value ranges ≥ 75% indicated. The genus *Trigastrotheca* had 100% bootstrap support. *Trigastrothecatridentata* was strongly supported (100% bootstrap) as sister group to *T.sureeratae* Quicke & Butcher, 2017, and three other Thai species (*T.doiphukhaensis* Raweearamwong, Quicke & Butcher, *T.khaoyaiensis* sp. nov. and *T.naniensis* sp. nov.) formed a clade with 96% bootstrap support, which, in turn, formed the sister group to the Australian *T.griffini* sp. nov. with 87% support.

### ﻿Key to the species of *Trigastrotheca* – not including males of Afrotropical species with entirely ochreous-yellow meso- and metasomas; for these see van Achterberg and Sigwalt (1987)

**Table:** 

1	Body unicolourous, mostly ochraceous yellow (Figs 2, 3, 5, 10, 14, 24, 33, 37)	**2**
–	Body bicolorous or at least metasoma bicolourous or tricolourous (combinations of black, red-brown, ochreous yellow and ivory white/cream) (Figs 7, 12, 17, 19, 21, 25, 27, 29)	**9**
2 (1)	Stemmaticum entirely yellow (Figs 3C, 5C)	**3**
–	Stemmaticum largely or entirely piceous or black (Figs 2A, 10C, 14C, 23E, 33D, 36D)	**4**
3 (2)	Fore wing vein C+SC+R black (Figs 3A, 4C, D) postero-lateral margin of T5 finely denticulate along lateral 0.75 (Fig. 4A); medial lobe of T5 forming obtuse angle (Fig. 4B); eye 2.5 × as long as temple in dorsal view. (Fig. 3C)	***T.braeti* Quicke & Butcher, sp. nov.**
–	Fore wing vein C+SC+R brown-yellow (Fig. 6A, B); postero-lateral margin of T5 distinctly more strongly denticulate on lateral 0.6 (Fig. 6A); medial lobe of T5 forming acute angle (Fig. 6C); eye 2.1 × as long as temple in dorsal view (Fig. 5C)	***T.carinata* Ranjith, sp. nov.**
4 (2)	T5 with medial lobe shorter than lateral lobes (Fig. 11B, C); postero-lateral margin of T5 unevenly serrate, denticles more developed laterally (Figs 11A, 24E)	**5**
–	T5 with medial lobe as long as lateral lobes; postero-lateral margin of T5 evenly serrate (Figs 4A, 33E, 37A, D)	**7**
5 (4)	Fore wing vein C+SC+R black (Fig. 24A, F); fore wing vein 3RSa > 1.4 × longer than r-rs (Fig. 24F)	***T.simba* van Noort, sp. nov.**
–	Fore wing vein C+SC+R brown-yellow (Figs 11D, 14A, 16B); fore wing vein 3RSa < 1.2 × longer than r-rs (Figs 11D, 16B)	**6**
6 (5)	OOL 2.0 × POL (Fig. 10C); propodeum without longitudinal striae posteriorly (Fig. 10F); fore wing vein 2RS 1.0 × as long as r-rs (Fig. 11D)	***T.flava* Ranjith, sp. nov.**
–	OOL > 2.0 × POL (Fig. 14C); propodeum with distinct longitudinal striae posteriorly (Fig. 15C); fore wing vein 2RS > 1.0 × longer than r-rs (Fig. 15B)	***T.freidbergi* Quicke & Friedman, sp. nov.**
7 (4)	Frons and vertex granulate (Fig. 32D); area of frons anterolateral to median ocellus with weakly diverging striae	***T.trilobata* Cameron, 1906**
–	Frons and vertex rugose (Figs 2C, 33D); area of frons anterolateral to median ocellus without striae	**8**
8 (7)	Second metasomal suture evenly wide and crenulated and weakly arched (Fig. 33F); lateral lobes of T5 rounded apically (Fig. 33E)	***T.romani* Quicke, 2005**
–	Second metasomal suture wider and more coarsely crenulated medially and distinctly arched (Fig. 2G); lateral lobes of T5 distinctly acute, spine like (Fig. 2E)	***T.aethiopica* Quicke & Friedman, sp. nov.**
9 (1)	Occiput yellow	**10**
–	Occiput piceous or black	**15**
10 (9)	T2 yellow	**11**
–	T2 dark brown or black medially surrounded by paler marks or at least black medially	**13**
11 (10)	Mesoscutum yellow without dark patches; TT1 and 2 with sublateral brownish patches; semicircular emargination of T5 yellow posteriorly	***T.luzonensis* Quicke & Butcher, 2017**
–	Mesoscutum yellow with dark patches; TT1 and 2 yellow or ivory white; semicircular emargination of T5 ivory white posteriorly	**12**
12 (11)	Antenna with 41 flagellomeres; notauli distinct, impressed; fore wing veins r-rs and 2RS straight; frons ivory white laterally	***T.tricolor* Quicke & Ingram, 1993**
–	Antenna with at least 51 flagellomeres; notauli virtually absent; fore wing veins r-rs and 2RS wavy; frons yellow laterally	***T.pariyanonthae* Quicke & Butcher, 2017**
13 (10)	Propodeum black; mesopleuron largely black; scutellum medially black with lateral yellowish spots; T2 with inverted T-shaped blackish patch	***T.doiphukhaensis* Raweearamwong, Quicke & Butcher, 2020**
–	Propodeum yellow; mesopleuron yellow; scutellum yellow; T2 almost completely blackish	**14**
14 (13)	Median area of metanotum with a distinct mid-longitudinal carina; base of hind wing with a well-developed glabrous area distal to vein cu-a; antenna with 49–53 flagellomeres; fore wing length > 4.5 [4.9–5.1] mm	***T.sureeratae* Quicke & Butcher, 2017**
–	Median area of metanotum without mid-longitudinal carina; base of hind wing with reduced setosity but this extending to vein cu-a anteriorly and with only a small glabrous area posteriorly; antennae with 45–47 flagellomeres; fore wing length < 4.0 [3.6] mm	***T.maetoi* Quicke & Butcher, 2017**
15 (9)	Mesoscutum reddish yellow or dark chestnut red; without black patches on lateral lobes and on anterior of medial lobe (Figs 8B, 17E, 29D)	**16**
–	Mesoscutum with three large black patches on lateral lobes and anterior 1/2 of medial lobe (Figs 19D, 21E, 34E)	**19**
16 (15)	Hind femur and tibia reddish yellow (Figs 8E, 12E); face uniformly yellow or cream colored without black triangular mark above clypeus (Fig. 12B)	**17**
–	Hind femur and tibia reddish black (Figs 17A, 29A); face with black triangular mark above clypeus (Figs 17B, 29B)	**18**
17 (16)	Flagellum brown-yellow with apical 10 segments black (Fig. 7A); mesoscutum entirely dark chestnut red; scutellum medially black with whitish spots antero-laterally (Fig. 8B); postero-lateral emarginations of T5 shallow (Fig. 8D); mesopleuron with 2 black spots; occiput broadly black on upper 1/2 (Fig. 7C)	***T.christianhenrichi* Quicke & Butcher, sp. nov.**
–	Flagellum entirely black (Fig. 12A); mesoscutum with posterior 1/2 of middle lobe cream colored (Fig. 12E); scutellum completely yellow (Fig. 12E); postero-lateral emarginations of T5 deeply curved (Fig. 13A); mesopleuron with 1 black spot (Fig. 12D); occiput yellow (Fig. 12C)	***T.formosa* Quicke & Friedman, sp. nov.**
18 (16)	Metanotum with mid-longitudinal carina on anterior 1/2, bifurcated forming a large triangular area on posterior 1/2 (Fig. 30A); T2 with large black area medially (Fig. 30B); frons entirely black except with yellow spots antero-laterally (Fig. 29A)	***T.acroceropsis* Ranjith & Quicke, nom. nov.**
–	Metanotum with complete mid-longitudinal carina (Fig. 17E); T2 with narrow mid-longitudinal black stripe (Fig. 18B); frons black only medially (Fig. 17C)	***T.griffini* Quicke, sp. nov.**
19 (15)	Scutellum completely yellow without black patches (Fig. 27C); longitudinal black patch on middle lobe of mesoscutum present at anterior 1/2; antenna with 26–28 flagellomeres	**20**
–	Scutellum medially black, lateral margins yellow; longitudinal black patch on middle lobe of mesoscutum extending to posterior 1/2; antenna with > 30 flagellomeres	**22**
20 (19)	T5 of female with shallow sub-median semicircular emarginations and medial protuberance weak and very broad and not protruding beyond lateral lobes (Fig. 27E); frons without mid-longitudinal carina	***T.sublobata* Quicke, sp. nov.**
–	T5 of female with deep sub-median semicircular emarginations creating strong medial point that protrudes beyond lateral lobes (Fig. 35A); frons with mid-longitudinal carina	**21**
21 (20)	Mesopleuron with black patches; antenna with 26 or 27 flagellomeres; POL 1.2 × OOL	***T.laikipiensis* Quicke, 2005**
–	Mesopleuron without black patches; antenna with 38 flagellomeres; POL 0.4 × OOL	***T.sureeratae* Quicke & Butcher, 2017**
22 (19)	Face brownish ventrally and laterally (Fig. 25B); pronotum brownish laterally (Fig. 25D); occiput black laterally (Fig. 25C, D)	**23**
–	Face completely yellow (Fig. 19B); pronotum yellow except for at most a small blackish spot (Figs 19E, 21D); occiput yellow laterally (Figs 19C, 21C)	**24**
23 (22)	T2 with broad brown patch medially; postero-lateral margin of T5 concave laterally forming sharp angulation with lateral margin; T5 brown antero-laterally	***T.tridentata* (Enderlein, 1920)**
–	T2 with narrow piceous patch medially (Fig. 26C); postero-lateral margin of T5 weakly convex merging the lateral margin as smoothly in a curve (Fig. 26B); T5 yellow antero-laterally (Fig. 26D)	***T.similidentata* Ranjith, sp. nov.**
24 (22)	T4 thin and pale posteriorly (Fig. 20B); propodeum anterolaterally with cream-colored mark (Fig. 19E); median posterior lobe of T5 narrow, sides converging at an acute angle (Fig. 20B); OOL 2.1 × POL (Fig. 19C)	***T.khaoyaiensis* Quicke & Butcher, sp. nov.**
–	T4 black; posteriorly; propodeum entirely black (Fig. 21D); median posterior lobe of T5 broadly rounded, sides converging at an obtuse angle (Fig. 22D); OOL 1.7 × POL (Fig. 21C)	***T.naniensis* Quicke & Butcher, sp. nov.**

### ﻿Descriptive taxonomy

#### ﻿ Trigastrotheca aethiopica

Taxon classificationAnimaliaHymenopteraBraconidae

Quicke & Friedman sp. nov.

90691BA6-ED23-55E5-B833-D195E969C1CB

https://zoobank.org/DA6BED39-C1C9-467E-9C6C-0CFE555CC2B0

Fig. 2

##### Type material.

***Holotype*** ♀, Ethiopia, Erer River, Rt.4, 20 km E. Harar, 9°14.5'N, 42°14.8'E, 11.ix.2007, 1330 m, coll. L. Friedman (SMNHTAU).

##### Diagnosis.

**Figure 2.**
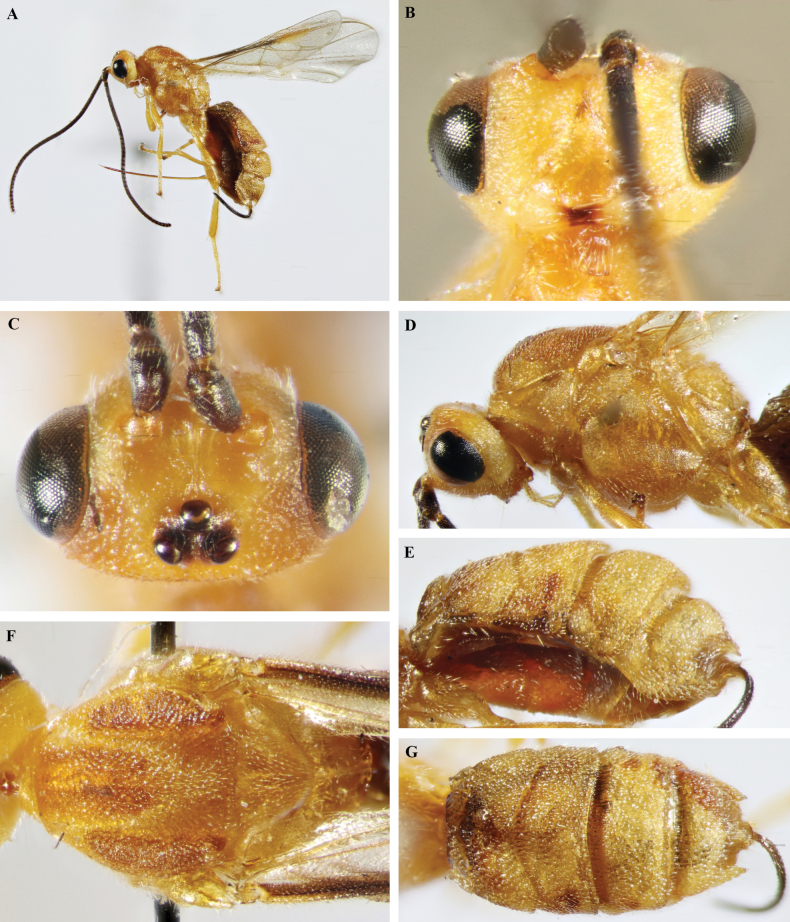
*Trigastrothecaaethiopica* sp. nov. ♀, holotype **A** habitus, lateral view **B** head, anterior view **C** head, dorsal view **D** head and mesosoma, lateral view **E** metasoma, lateral view **F** mesosoma, dorsal view **G** metasoma, dorsal view.

Body ochreous yellow except for piceous stemmaticum. Similar to *T.trilobata* and *T.romani*. Differs from *T.trilobata* in having the anterior of the pterostigma yellow (Fig. 2A). Differs from *T.romani* in having the anterior of pterostigma yellow and the second metasomal suture more strongly arched and distinctly wider medially (Fig. 2G cf. Fig. 33F).

##### Description.

Holotype female. Length of body 4.9 mm, fore wing 4.6 mm. ***Head*.** Antenna incomplete with 39 flagellomeres. Terminal flagellomere lost. First flagellomere 1.2 × longer than 2^nd^ and 3^rd^, the latter 1.8 × longer than wide. Width of head: width of face: height of eye = 2.5: 1.5: 1.0. Face with fine transverse striations laterally; with weak mid-longitudinal ridge. Inter-tentorial distance 1.5 × longer than tentorio-ocular distance. Malar suture impressed. Malar space 1.3 × as long as basal width of mandible. Frons strongly impressed with a complete mid-longitudinal carina. Shortest distance between posterior ocelli: transverse diameter of posterior ocellus: shortest distance between posterior ocellus and eye = 1.0: 1.0: 2.5. ***Mesosoma*** 1.4 × longer than high. Mesoscutum rugose; notauli not impressed except very short anterior part with few weak crenulations, very shallow posteriorly. Scutellar sulcus shallow, narrow, finely crenulate. Scutellum smooth, setose without small pit medially behind sulcus. Median area of metanotum with complete mid-longitudinal carina. Propodeum largely smooth and shiny; mid-longitudinal carina complete, lamelliform, bordered narrowly by fine crenulations; posteriorly propodeum with short longitudinal carinae associated with longitudinal wrinkles. ***Wings*.** Fore wing. Lengths of fore wing veins r-rs: 3RSa: 3RSb = 1.0: 1.5: 4.7. Lengths of vein 2RS: 3RSa: rs-m = 1.5: 1.5: 1.0. Base of hind wing glabrous. ***Legs*.** Lengths of fore femur: fore tibia: fore tarsus = 1.0: 1.3: 1.3. Lengths of hind femur: hind tibia: hind tarsus = 1.0: 1.2: 1.3. Claws with small acutely pointed basal lobe. ***Metasoma*.** T1 1.6 × wider than long. T2 0.8 × as long as T3. T1 coriaceous. TT1–5 with coarse reticulate sculpture. Second metasomal suture and basal grooves of TT4 and 5 deep, strigose. T5 with postero-lateral margin of convex, distinctly and evenly denticulate; medial protuberance acutely rounded. ***Coloration*.** Body mostly yellow except antenna, eye and tarsi black, and stemmaticum which is dark brown.

**Male.** Unknown.

##### Distribution.

Afrotropical (Ethiopia).

##### Host.

Unknown.

##### Etymology.

Specific name refers to Ethiopia, the provenance of the holotype.

#### ﻿ Trigastrotheca braeti

Taxon classificationAnimaliaHymenopteraBraconidae

Quicke & Butcher sp. nov.

3401E458-7762-50B1-AF6B-70A4D5BD45A0

https://zoobank.org/0D16308C-DF02-42B2-A85E-8F46DDF6064E

Figs 3 , 4

##### Type material.

***Holotype*** ♀, The Republic of the Congo, Pool, Abio Lesio-Louna N.P., 1.vi–18.vii.2008, 3.099125°S, 15.27157°W, 330 m, Malaise trap in forest-savanna transition zone, coll. Yves Braet, DNA voucher P.I.D. BBTH4962-22 (CUMZ). ***Paratypes***: 1 ♀, same data as holotype except 23.x.2008, coll. Y. Braet & M.J. Sharkey, DNA voucher P.I.D. BBTH1561-18 (failed); 1 ♂, same data as holotype except 3°16.1965'S, 15°28.267'E, 23.x.2008, coll. coll. Y. Braet & M.J. Sharkey, DNA voucher P.I.D. BBTH1557-18 (failed) (CUMZ).

##### Diagnosis.

Similar to *T.carinata* sp. nov. from India, in having the body including stemmaticum entirely ochreous yellow, but differs in the shape of the head with the eye 2.5 × as long as temple in dorsal view. In addition, fore wing vein C+SC+R black, fore wing veins r-rs and rs-m wavy, and the T5 is relatively more elongate but with the posterior emarginations being shallower and medio-posterior projection being less acute.

##### Description.

Holotype female. Length of body 5.0 mm, fore wing 4.3 mm. ***Head*.** Antenna with 41 flagellomeres. Terminal flagellomere, short, sub-triangular, acuminate. First flagellomere 1.0 × longer than 2^nd^ and 3^rd^, the latter 1.4 × longer than wide. Width of head: width of face: height of eye = 2.5: 1.5: 1.0. Face granulate rugose, transversely striate antero-medially with weak mid-longitudinal ridge. Inter-tentorial distance 1.3 × longer than tentorio-ocular distance. Malar suture impressed. Malar space 1.7 × as long as basal width of mandible. Antennal sockets strongly produced. Frons rugose with a mid-longitudinal groove, strongly moderately behind antennal socket. Shortest distance between posterior ocelli: transverse diameter of posterior ocellus: shortest distance between posterior ocellus and eye = 1.3: 1.0: 2.8. ***Mesosoma*** 1.3 × longer than high. Mesoscutum rugose; notauli not impressed except very short anterior part with few weak crenulations. Scutellar sulcus shallow, narrow, finely crenulate. Scutellum granulate with a small pit medially behind sulcus. Median area of metanotum with complete mid-longitudinal carina. Propodeum rugose; mid-longitudinal carina complete, lamelliform, bordered narrowly by fine crenulations; posteriorly propodeum with short longitudinal carinae associated with longitudinal wrinkles. ***Wings*.** Fore wing. Lengths of fore wing veins r-rs: 3RSa: 3RSb = 1.0: 1.2: 4.7. Lengths of vein 2RS: 3RSa: rs-m = 1.1: 1.0: 1.1. Base of hind wing with short glabrous area. ***Legs*.** Lengths of fore femur: fore tibia: fore tarsus = 1.0: 1.0: 1.1. Lengths of hind femur: hind tibia: hind tarsus = 1.0: 1.3: 1.2. Claws with basal lobe rather small, distally angulate but hardly protruding. ***Metasoma*.** T1 1.5 × wider than long. T2 0.9 × as long as T3. T1 coriaceous. TT1–5 with coarse reticulate sculpture Second metasomal suture and basal grooves of TT4 and 5 deep, strigose. T5 with postero-lateral margin of convex, indistinctly denticulate; medial protuberance broadly rounded. ***Coloration*.** Body yellow except scape, pedicel, pterostigma, tarsi brown, flagellomere yellowish brown.

**Figure 3.**
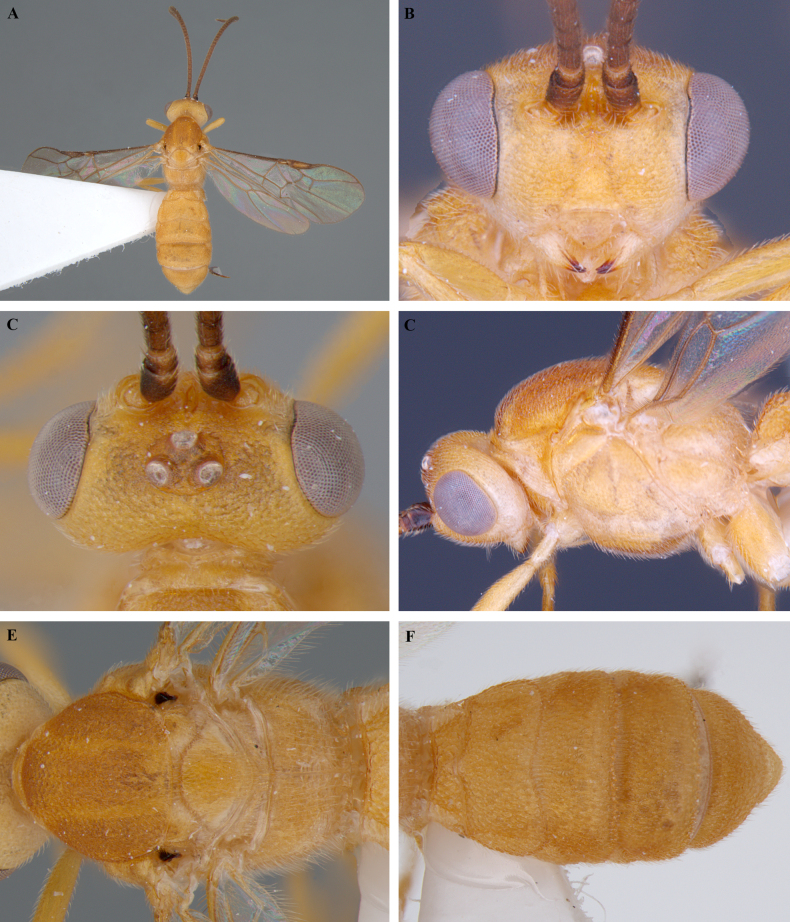
*Trigastrothecabraeti* sp. nov. ♀, holotype **A** habitus, dorsal view **B** head, anterior view **C** head, dorsal view **D** head and mesosoma, lateral view **E** mesosoma, dorsal view **F** metasoma, dorsal view.

**Figure 4.**
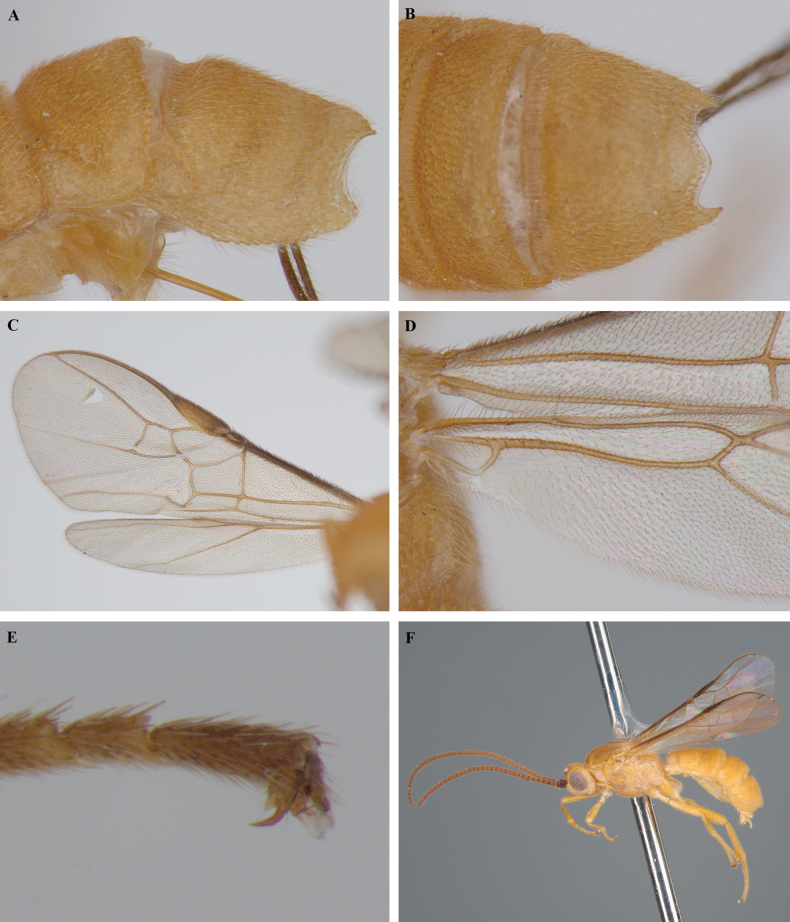
*Trigastrothecabraeti* sp. nov. ♀, holotype **A** TT4 and 5, lateral view **B** TT4 and 5, dorsal view **C** wings **D** base of hind wing, detail **E** apex of hind tarsus and claw; Paratype ♂ **F** habitus, lateral view.

**Variation**. Body length 4.6 mm paratype female, 4.4 mm paratype male. Posterior margin of male T5 virtually straight with a weak medial emargination.

**Male.** Same as female.

##### Distribution.

Afrotropical (Republic of Congo).

##### Host.

Unknown.

##### Etymology.

Named after Dr Yves Braet, collector and friend of the senior author.

#### ﻿ Trigastrotheca carinata

Taxon classificationAnimaliaHymenopteraBraconidae

Ranjith sp. nov.

53383AE5-03E4-5572-96E5-7A6A01DAB299

https://zoobank.org/F747A595-E9F5-4EE3-A958-CE3EBD047B2A

Figs 5 , 6

##### Type material.

***Holotype*** ♀, India: Kerala, Palakkad, Silent Valley, 17.iv.2018, Malaise trap, coll. Sinu P.A. (AIMB).

##### Diagnosis.

Similar to *T.braeti* sp. nov. from Africa, in having the body including stemmaticum entirely ochreous yellow, but differs in the shape of the head with the eye only 2.1 × as long as temple in dorsal view. In addition, fore wing vein C+SC+R brown-yellow, fore wing veins r-rs and rs-m are not wavy, and the T5 is relatively broader with deeper posterior emarginations and a more acute medio-posterior projection. Similar also to *T.freidbergi* sp. nov. but stemmaticum yellow and T5 relatively far longer (Fig. 6C cf. Fig. 16A).

##### Description.

Holotype female. Length of body 4.0 mm, fore wing 3.0 mm. ***Head*.** Antenna with 42 flagellomeres. Terminal flagellomere, short, sub-triangular, acuminate. First flagellomere 1.0 × longer than 2^nd^ and 3^rd^, the latter 1.3 × longer than wide. Width of head: width of face: height of eye = 2.5: 1.4: 1.0. Face transversely striate-rugose; with weak mid-longitudinal ridge. Inter-tentorial distance 1.4 × longer than tentorio-ocular distance. Malar suture impressed. Malar space 2.1 × as long as basal width of mandible. Antennal sockets strongly produced. Frons strongly impressed behind antennal sockets, with mid-longitudinal carina. Shortest distance between posterior ocelli: transverse diameter of posterior ocellus: shortest distance between posterior ocellus and eye = 1.0: 1.0: 2.1. ***Mesosoma*** 1.4 × longer than high. Mesoscutum rugose; notauli not impressed except very short anterior part with few weak crenulations. Scutellar sulcus shallow, narrow, finely crenulate, very shallow posteriorly. Scutellum sparsely punctate, setose without small pit medially behind sulcus. Median area of metanotum with complete mid-longitudinal carina. Propodeum faintly rugose; mid-longitudinal carina complete, lamelliform, bordered narrowly by fine crenulations; posteriorly propodeum with short longitudinal carinae associated with longitudinal wrinkles. ***Wings*.** Fore wing. Lengths of fore wing veins r-rs: 3RSa: 3RSb = 1.0: 1.3: 4.5. Lengths of vein 2RS: 3RSa: rs-m = 1.0: 1.1: 1.0. Base of hind wing with sparse setae. ***Legs*.** Lengths of fore femur: fore tibia: fore tarsus = 1.1: 1.2: 1.0. Lengths of hind femur: hind tibia: hind tarsus = 2.0: 2.3: 1.0. Claws with small acutely pointed basal lobe. ***Metasoma*.** T1 2.8 × wider than long. T2 1.1 × as long as T3. T1 coriaceous. TT1–5 with coarse reticulate sculpture. Second metasomal suture and basal grooves of TT4 and 5 deep, strigose. T5 with postero-lateral margin of convex, unevenly denticulate; medial protuberance acutely rounded posteriorly. ***Coloration*.** Body mostly yellow except antenna, apex of mandible, tarsi, ovipositor sheath brown.

**Figure 5.**
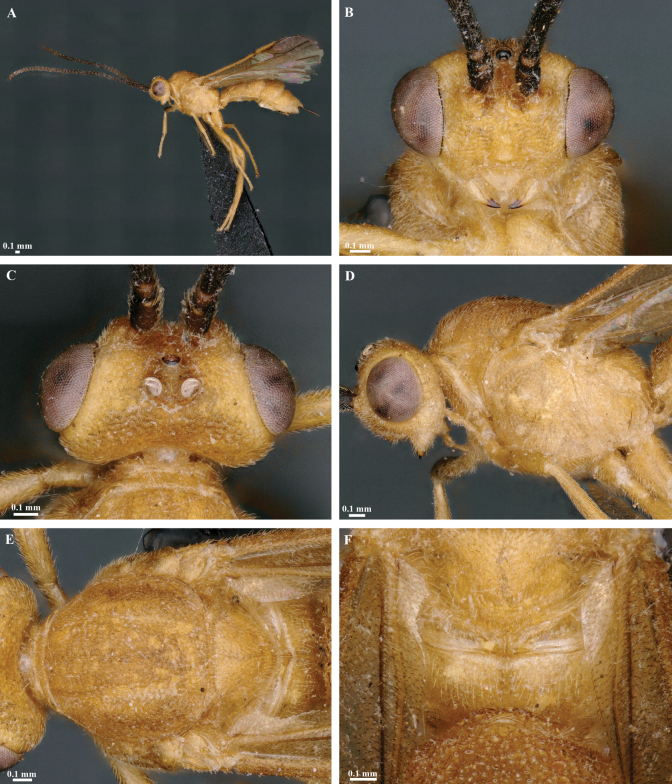
*Trigastrothecacarinata* Ranjith, sp. nov. ♀ holotype **A** habitus, lateral view **B** head, anterior view **C** head, dorsal view **D** head and mesosoma, lateral view **E** mesosoma, dorsal view **F** scutellum, metanotum and propodeum, dorsal view.

**Figure 6.**
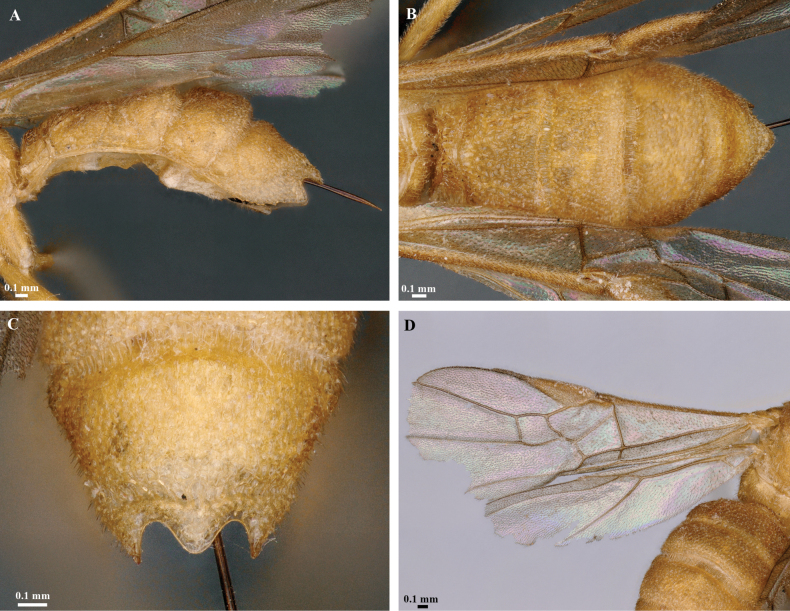
*Trigastrothecacarinata* Ranjith, sp. nov. ♀ holotype **A** metasoma, lateral view **B** mesosoma, dorsal view **C** T5, dorsal view **D** wings.

**Male.** Unknown.

##### Distribution.

Oriental (India).

##### Host.

Unknown.

##### Etymology.

The species is named after the presence of mid-longitudinal carina of frons which will separate the species from other Indian species.

#### ﻿ Trigastrotheca christianhenrichi

Taxon classificationAnimaliaHymenopteraBraconidae

Quicke & Butcher sp. nov.

983902FB-09F6-5D91-81E9-00935DAB900A

https://zoobank.org/1BD13FD7-E056-4E2F-B254-C25074FEC1C8

Figs 7 , 8 , 9

##### Type material.

***Holotype*** ♀, Madagascar, Fianarantsoa Province, Parc National Ranomafana, Malaise trap in mixed tropical forest, radio tower at forest edge, 21°15.05'S, 47°24.43'E, 1130 m, 21–28.i.2002, col. R. Harin’Hala, (CALACAD).

##### Diagnosis.

**Figure 7.**
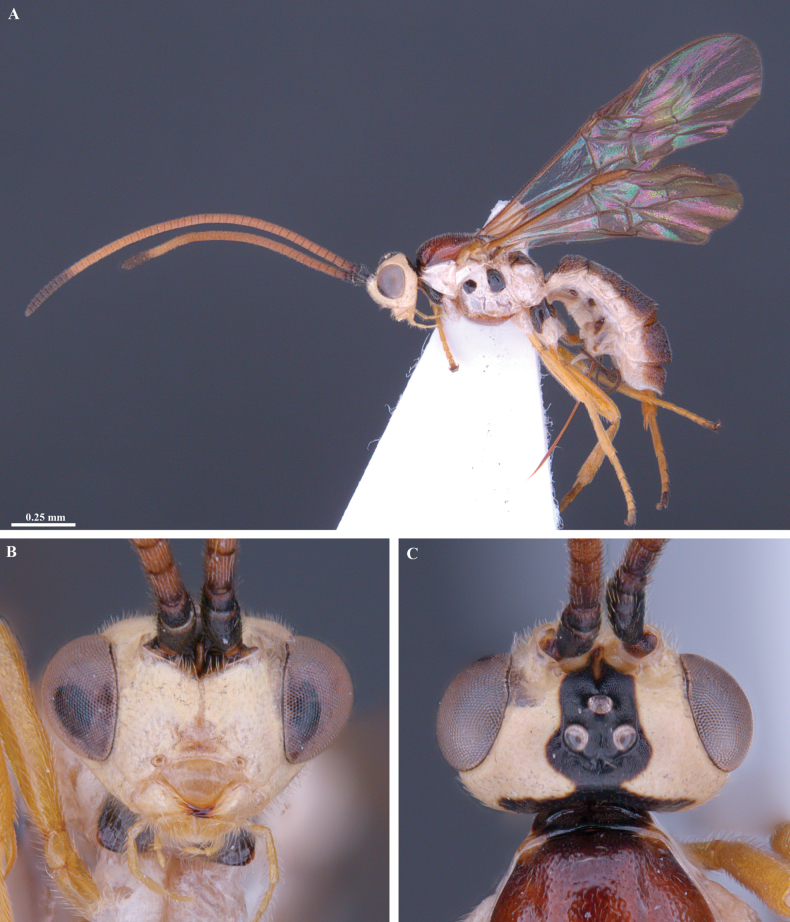
*Trigastrothecachristianhenrichi* sp. nov. ♀ holotype **A** habitus, lateral view **B** head, anterior view **C** head, dorsal view.

This is the only species with bicolorous antennal flagellum, mostly orange-yellow but with approximately apical 10 black (Fig. 7A). Otherwise with a largely similar color pattern to *T.formosa* sp. nov. though darker red-brown (chestnut), both having a largely red mesoscutum. Unlike in the latter, the back of the head has a wide black mark and the mesoscutum is uniformly red-brown.

##### Description.

Length of body 5.0 mm, fore wing 5.3 mm. ***Head*.** Antenna with 50 flagellomeres. Terminal flagellomere, short, sub-triangular, acuminate. First flagellomere 1.1 × longer than 2^nd^ and 3^rd^, the latter 1.2 × longer than wide. Width of head: width of face: height of eye = 2.1: 1.1: 1.0. Face granulate; with weak mid-longitudinal ridge. Inter-tentorial distance 1.7 × longer than tentorio-ocular distance. Malar suture impressed. Malar space 1.5 × as long as basal width of mandible. Antennal sockets strongly produced, laterally, lamelliform, giving rise posteriorly to a longitudinal carina that divides the anterior frons into four depressed pits, two laterally and two on the sides of carina; lateral to these carinae frons strongly impressed, pit like. Frons, vertex, and occiput granulate. Shortest distance between posterior ocelli: transverse diameter of posterior ocellus: shortest distance between posterior ocellus and eye = 1.5: 1.0: 3.0. ***Mesosoma*** 1.5 × longer than high. Mesoscutum smooth, sparsely punctate; notauli not impressed except very short anterior part with few weak crenulations, very shallow posteriorly. Scutellar sulcus shallow, narrow, finely crenulate. Scutellum smooth, sparsely setose with a small pit medially behind sulcus. Median area of metanotum with complete mid-longitudinal carina. Propodeum largely smooth and shiny, faintly granulate near mid-longitudinal carina; mid-longitudinal carina complete, lamelliform, bordered narrowly by fine crenulations; posteriorly propodeum with short longitudinal carinae associated with longitudinal wrinkles. ***Wings*.** Fore wing. Lengths of fore wing veins r-rs: 3RSa: 3RSb = 1.0: 1.1: 4.3. Lengths of vein 2RS: 3RSa: rs-m = 1.3: 1.5: 1.0. Base of hind wing with large glabrous area distal to vein 1CU. ***Legs*.** Lengths of fore femur: fore tibia: fore tarsus = 1.3: 1.0: 1.2. Lengths of hind femur: hind tibia: hind tarsus = 1.0: 1.1: 1.2. Claws with small acutely pointed basal lobe. ***Metasoma*.** T1 coriaceous, 3.3 × wider than long with a pair of posteriorly narrowing dorsal carina. T2 1.1 × as long as T3. TT1–5 with coarse reticulate sculpture. Second metasomal suture and basal grooves of TT4 and 5 deep, strigose. T5 with postero-lateral margin of convex, not denticulate; medial protuberance rounded posteriorly; postero-lateral emarginations not defined. ***Coloration*.** Antenna largely reddish brown, scape and pedicel, terminal 10 flagellomeres black. Head ochraceous yellow except for large black, T-shaped mark around stemmaticum and back of head. Mesoscutum reddish brown except blackish anteriorly, pronotum dorsally, propleuron black, pronotum laterally, scutellum antero-laterally and posteriorly, mesopleuron except a pair of black patches, metapleuron, posterior 1/2 of propodeum, fore, mid, and distal 1/2 of hind coxa, metasoma laterally, posterior margin of T5 ivory white, propodeum anterior 1/2, metasoma except laterally reddish brown.

**Figure 8.**
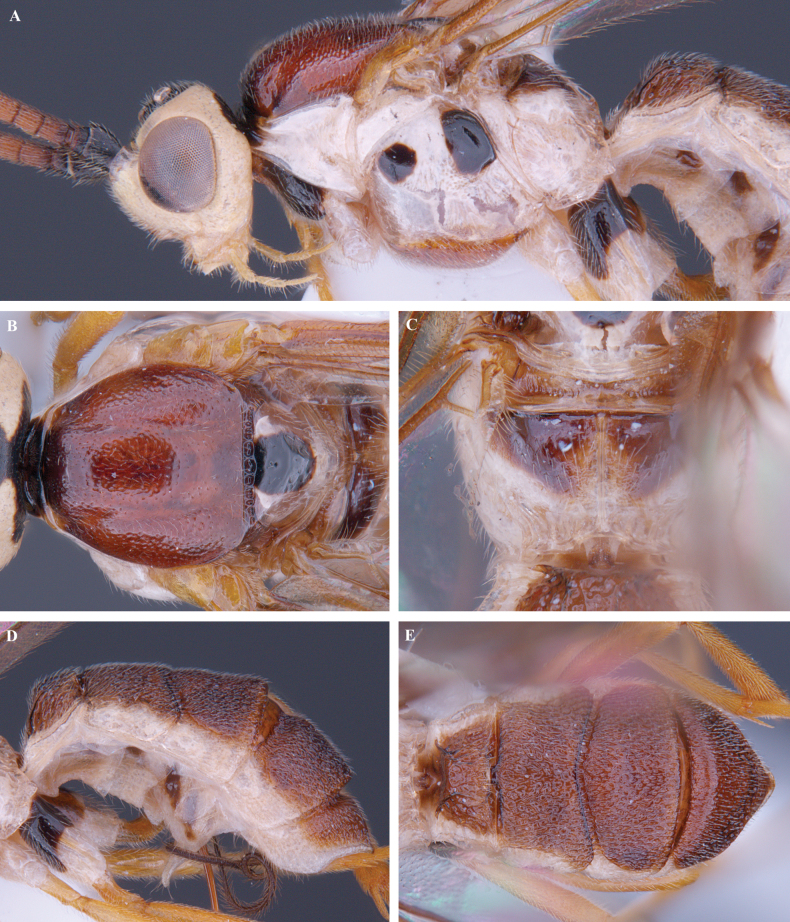
*Trigastrothecachristianhenrichi* sp. nov. ♀ holotype **A** habitus, except posterior metasoma, lateral view **B** mesosoma, dorsal view **C** metanotum and propodeum, dorsal view **D** metasoma, lateral view **E** mesosoma, dorsal view.

**Figure 9.**
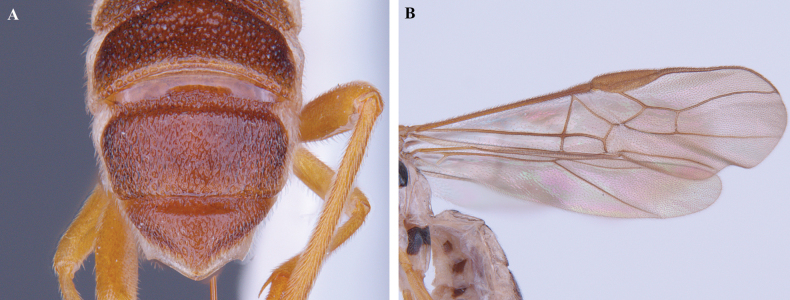
*Trigastrothecachristianhenrichi* sp. nov. ♀ holotype **A** TT3–5, dorsal view **B** wings.

**Male.** Unknown.

##### Distribution.

Afrotropical (Madagascar).

##### Host.

Unknown.

##### Etymology.

Named after the senior author’s friend Christian Henrich.

#### ﻿ Trigastrotheca flava

Taxon classificationAnimaliaHymenopteraBraconidae

Ranjith sp. nov.

6843C484-F42E-508D-BE81-BC86ADBE1AFF

https://zoobank.org/6E22C497-8E35-40FE-AC81-2284C644C3EF

Figs 10 , 11

##### Type material.

***Holotype*** ♀, India: Karnataka, Chintamani, 26.v.2010, coll. Somraj Gunda (AIMB). ***Paratypes*.** 4 ♀ with the same data as holotype (AIMB).

##### Diagnosis.

Similar to *Trigastrothecafreidbergi* Quicke & Friedman, sp. nov. in being entirely ochraceous with piceous/black stemmaticum and brown-yellow fore wing vein C+SC+R and fore wing with a short 2^nd^ submarginal cell (vein 3RSa being < 1.2 × longer than r-rs and shorter than 2RS).

##### Description.

Holotype ♀. Length of body 4.3 mm, fore wing 3.4 mm. ***Head*.** Antenna with 39 flagellomeres. Terminal flagellomere, short, sub-triangular, acuminate. First flagellomere 1.0 × longer than 2^nd^ and 3^rd^, the latter 1.2 × longer than wide. Width of head: width of face: height of eye = 2.8: 1.5: 1.0. Face finely rugose-punctate with weak mid-longitudinal ridge. Inter-tentorial distance 1.8 × longer than tentorio-ocular distance. Malar suture impressed. Malar space 1.3 × as long as basal width of mandible. Antennal sockets not strongly produced. Frons moderately impressed without mid-longitudinal carina. Shortest distance between posterior ocelli: transverse diameter of posterior ocellus: shortest distance between posterior ocellus and eye = 1.1: 1.0: 2.7. ***Mesosoma*** 1.4 × longer than high. Mesoscutum rugose; notauli not impressed except very short anterior part with few weak crenulations. Scutellar sulcus shallow, narrow, finely crenulate. Scutellum finely punctate without small pit medially behind sulcus. Median area of metanotum with complete mid-longitudinal carina. Propodeum finely granulate-rugose; mid-longitudinal carina complete, faintly lamelliform, bordered narrowly by fine crenulations; posteriorly propodeum with short longitudinal carinae associated with longitudinal wrinkles. ***Wings*.** Fore wing. Lengths of fore wing veins r-rs: 3RSa: 3RSb = 1.0: 1.2: 3.9. Lengths of vein 2RS: 3RSa: rs-m = 1.3: 1.0: 1.0. Base of hind wing with at least a pair of setae. ***Legs*.** Lengths of fore femur: fore tibia: fore tarsus = 1.0: 1.1: 1.1. Lengths of hind femur: hind tibia: hind tarsus = 2.1: 2.3: 1.0. Claws with small acutely pointed basal lobe. ***Metasoma*.** T1 2.1 × wider than long. T2 0.9 × as long as T3. T1 coriaceous. TT1–5 with coarse reticulate sculpture. Second metasomal suture and basal grooves of TT4 and 5 deep, strigose. T5 with postero-lateral margin of convex, unevenly denticulate; medial protuberance acutely rounded posteriorly. ***Coloration*.** Body mostly yellow except antenna, apex of mandible, ovipositor sheath brown.

**Figure 10.**
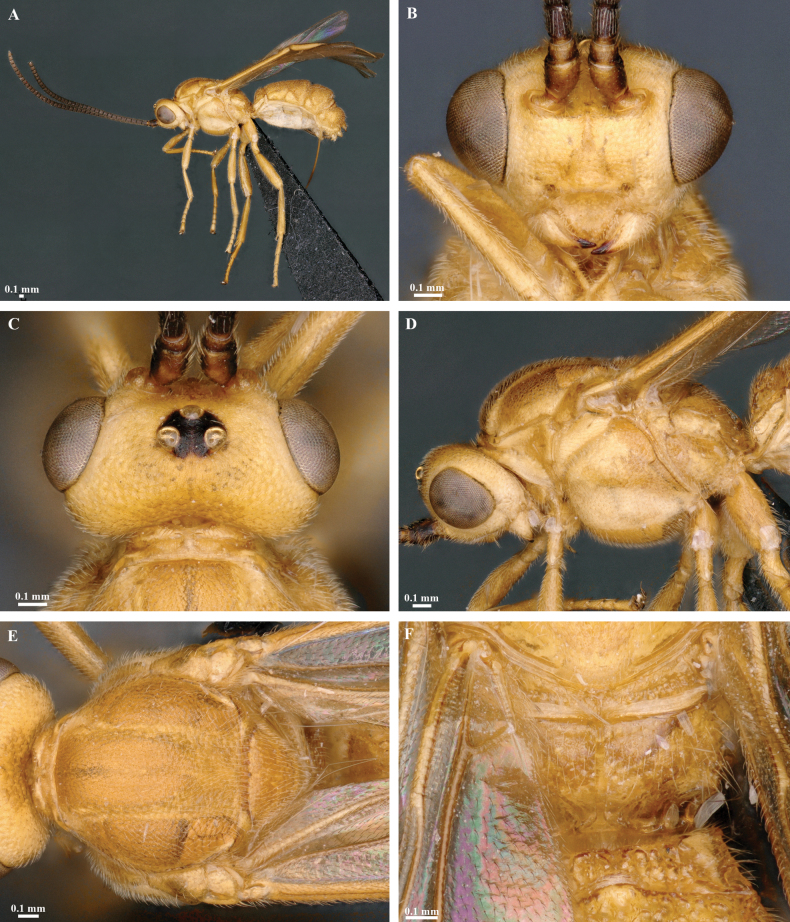
*Trigastrothecaflava* sp. nov. ♀ holotype **A** habitus, lateral view **B** head, anterior view **C** head, dorsal view **D** head and mesosoma, lateral view **E** mesosoma, dorsal view **F** metanotum and propodeum, dorsal view.

**Figure 11.**
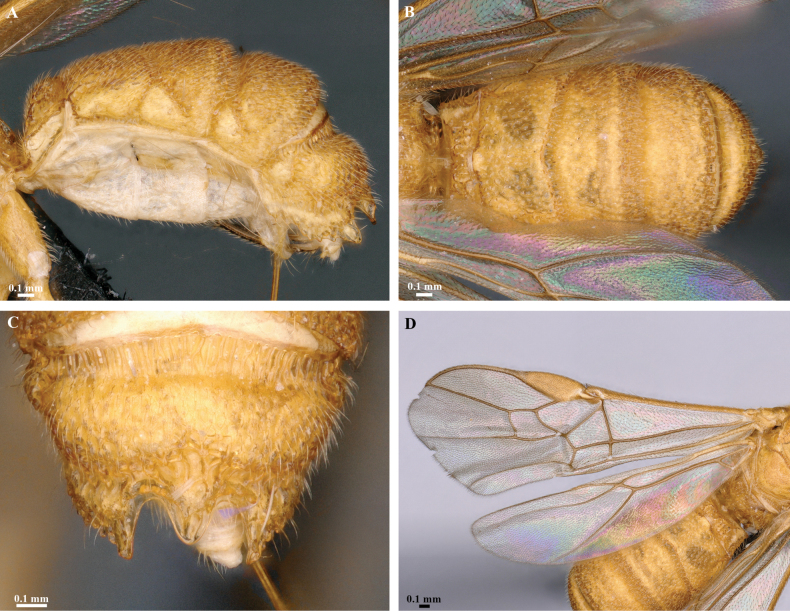
*Trigastrothecaflava* sp. nov. ♀ holotype **A** metasoma, lateral view **B** metasoma, dorsal view **C** T5, dorsal view **D** wings.

**Male.** Unknown.

##### Distribution.

Oriental (India).

##### Host.

Unknown.

##### Etymology.

The species is named after the yellow-colored body, *flava* meaning yellow in Latin.

#### ﻿ Trigastrotheca formosa

Taxon classificationAnimaliaHymenopteraBraconidae

Quicke & Friedman sp. nov.

E99037A0-6685-5648-9369-C2ACFD00084F

https://zoobank.org/91E3C3CA-0334-47CD-8F8C-7E5EF5A66BF5

Figs 12 , 13

##### Type material.

***Holotype*** ♀, Madagascar, Analamatsaky, 47 km W Tolanaro, spiny forest, 25°1.0'S, 46°37.0'E, 21.x.2007, L. Friedman, DNA voucher P.I.D. BBTH744-17 (SMNHTAU). ***Paratypes***: 2 ♀, same data as holotype; one DNA voucher P.I.D. BBTH743-17 (SMNHTAU).

##### Diagnosis.

Similar to *T.christianhenrichi* sp. nov., also from Madagascar, but differing in having entirely dark flagellum, the black mark on the stemmaticum only extending to a point on the vertex and anterior occiput, the mesoscutum with posterior 1/2 of middle lobe cream-colored, and the scutellum reddish yellow.

##### Description.

Length of body 4.1 mm, fore wing 4.2 mm. ***Head*.** Antenna incomplete with 38 flagellomeres. Terminal flagellomere lost. First flagellomere 1.1 × longer than 2^nd^ and 3^rd^, the latter 1.5 × longer than wide. Width of head: width of face: height of eye = 2.4: 1.3: 1.0. Face granulate with weak mid-longitudinal ridge. Inter-tentorial distance 1.8 × longer than tentorio-ocular distance. Malar suture impressed. Malar space 1.5 × as long as basal width of mandible. Antennal sockets strongly produced. Frons strongly impressed only behind antennal sockets without mid-longitudinal carina. Frons, vertex, and occiput granulate. Shortest distance between posterior ocelli: transverse diameter of posterior ocellus: shortest distance between posterior ocellus and eye = 1.0: 1.0: 1.7. ***Mesosoma*** 1.5 × longer than high. Mesoscutum smooth, sparsely punctate; notauli crenulated, impressed on anterior 1/2 shallow posteriorly. Scutellar sulcus shallow, narrow, finely crenulate. Scutellum smooth, sparsely setose with a small pit medially behind sulcus. Median area of metanotum with complete mid-longitudinal carina. Propodeum largely smooth and shiny, faintly transversely striate near mid-longitudinal carina; mid-longitudinal carina complete, lamelliform, bordered narrowly by fine crenulations; posteriorly propodeum with short longitudinal carinae associated with longitudinal wrinkles. ***Wings*.** Fore wing. Lengths of fore wing veins r-rs: 3RSa: 3RSb = 1.0: 1.2: 4.0. Lengths of vein 2RS: 3RSa: rs-m = 1.3: 1.3: 1.0. Base of hind wing with large glabrous area distal to vein 1CU. ***Legs*.** Lengths of fore femur: fore tibia: fore tarsus = 1.0: 1.0: 1.1. Lengths of hind femur: hind tibia: hind tarsus = 1.0: 1.1: 1.1. Claws with small acutely pointed basal lobe. ***Metasoma*.** T1 coriaceous, 1.8 × wider than long, with pair of posteriorly uniting dorsal carina. T2 0.8 × as long as T3. TT1–5 with coarse reticulate sculpture. Second metasomal suture and basal grooves of TT4 and 5 deep, strigose. T5 with postero-lateral margin slightly concave, not denticulate; medial protuberance acutely rounded posteriorly; postero-lateral emarginations concave. ***Coloration*.** Body mostly reddish brown except scape, pedicel, eye, ocellar area, occiput medially, mesoscutum antero-laterally, propleuron, mesopleuron with posterior black patch, T1 medially, ovipositor sheath black, face, clypeus, maxillary and labial palps, frons, vertex and occiput laterally, pronotum dorsally, legs yellow, pronotum laterally, middle lobe of mesoscutum posteriorly, mesopleuron anteriorly, metasoma laterally ivory white.

**Figure 12.**
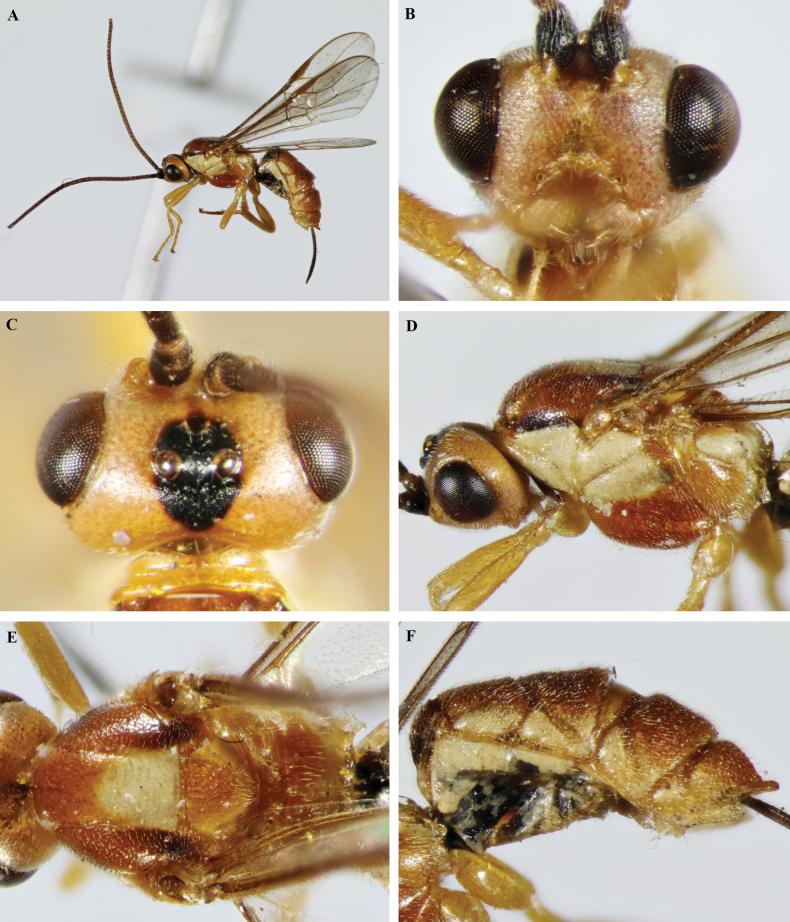
*Trigastrothecaformosa* sp. nov. ♀ holotype **A** habitus, lateral view **B** head, anterior view **C** head, dorsal view **D** head and mesosoma, lateral view **E** mesosoma, dorsal view **F** metasoma, lateral view.

**Figure 13.**
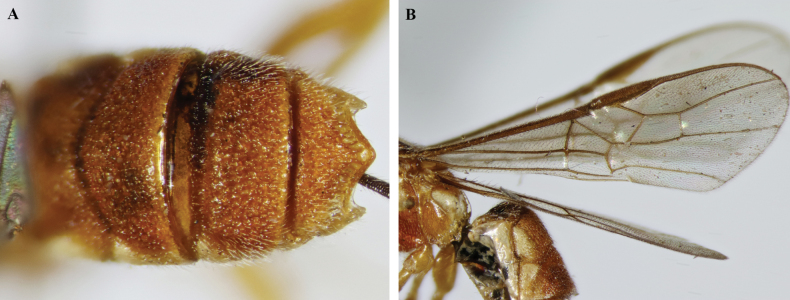
*Trigastrothecaformosa* sp. nov. ♀ holotype **A** TT3–5, dorsal view **B** wings.

**Male.** Unknown.

##### Distribution.

Afrotropical (Madagascar).

##### Host.

Unknown.

##### Etymology.

Latin, *formosa*, meaning beautiful.

##### Notes.

Excluded from the type series is a female with same data as the holotype but with the head entirely black but otherwise extremely similar. This seems to be a particularly unusual color variant for intraspecific variability, but it seems most probable, in the absence of additional data, that this is conspecific.

#### ﻿ Trigastrotheca freidbergi

Taxon classificationAnimaliaHymenopteraBraconidae

Quicke & Friedman sp. nov.

D6785DD8-D815-5261-99D4-6C38FBA29EBA

https://zoobank.org/BD2AD2C4-25E9-4C21-B355-F45AACA98F54

Figs 14 , 15 , 16

##### Type material.

***Holotype*** ♀, India: Rajasthan, Nagda Temple, 25 km N Udaipur Lake, 22.xi.2002, A. Freidberg (CUMZ). ***Paratype*.** 4 ♀, same data as holotype (2 in SMNHTAU, 2 in MNB).

##### Diagnosis.

Uniformly brownish yellow but with stemmaticum and fore wing vein C+SC+R and pterostigma pale brown-yellow, second submarginal cell short with vein 3RSa shorter that 2RS (Fig. 16B). Similar to *Trigastrothecacarinata* Ranjith, sp. nov. but stemmaticum black and T5 relatively far shorter (Fig. 16A cf. Fig. 6C).

##### Description.

Holotype female. Length of body 5.5 mm, fore wing 3.9 mm. ***Head*.** Antenna with 38 flagellomeres. Terminal flagellomere acuminate. First flagellomere 1.2 × longer than 2^nd^ and 3^rd^, the latter 1.5 × longer than wide. Width of head: width of face: height of eye = 2.5: 1.3: 1.0. Face with fine transverse striations laterally; with weak mid-longitudinal ridge. Inter-tentorial distance 1.8 × longer than tentorio-ocular distance. Malar suture impressed. Malar space 1.2 × as long as basal width of mandible. Antennal sockets strongly produced. Frons strongly impressed with a complete mid-longitudinal carina. Shortest distance between posterior ocelli: transverse diameter of posterior ocellus: shortest distance between posterior ocellus and eye = 1.0: 1.0: 2.0. ***Mesosoma*** 1.4 × longer than high. Mesoscutum rugose; notauli not impressed except very short anterior part with few weak crenulations. Scutellar sulcus shallow, narrow, finely crenulate. Scutellum sparsely punctate without small pit medially behind sulcus. Median area of metanotum with complete mid-longitudinal carina. Propodeum largely smooth and shiny, faintly rugose antero-laterally; mid-longitudinal carina complete, not lamelliform, bordered narrowly by fine crenulations; posteriorly propodeum with short longitudinal carinae. ***Wings*.** Fore wing. Lengths of fore wing veins r-rs: 3RSa: 3RSb = 1.0: 1.2: 4.5. Lengths of vein 2RS: 3RSa: rs-m = 1.1: 1.0: 1.1. Base of hind wing glabrous. ***Legs*.** Lengths of fore femur: fore tibia: fore tarsus = 1.0: 1.1: 1.1. Lengths of hind femur: hind tibia: hind tarsus = 1.0: 1.2: 1.1. Claws with small acutely pointed basal lobe. ***Metasoma*.** T1 1.7 × wider than long. T2 0.9 × as long as T3. T1 coriaceous. TT1–5 with coarse reticulate sculpture Second metasomal suture and basal grooves of TT4 and 5 deep, strigose. T5 with postero-lateral margin of convex, unevenly denticulate; medial protuberance acutely rounded. ***Coloration*.** Body mostly yellow except antenna, eye, stemmaticum, tarsi, ovipositor sheath black.

**Figure 14.**
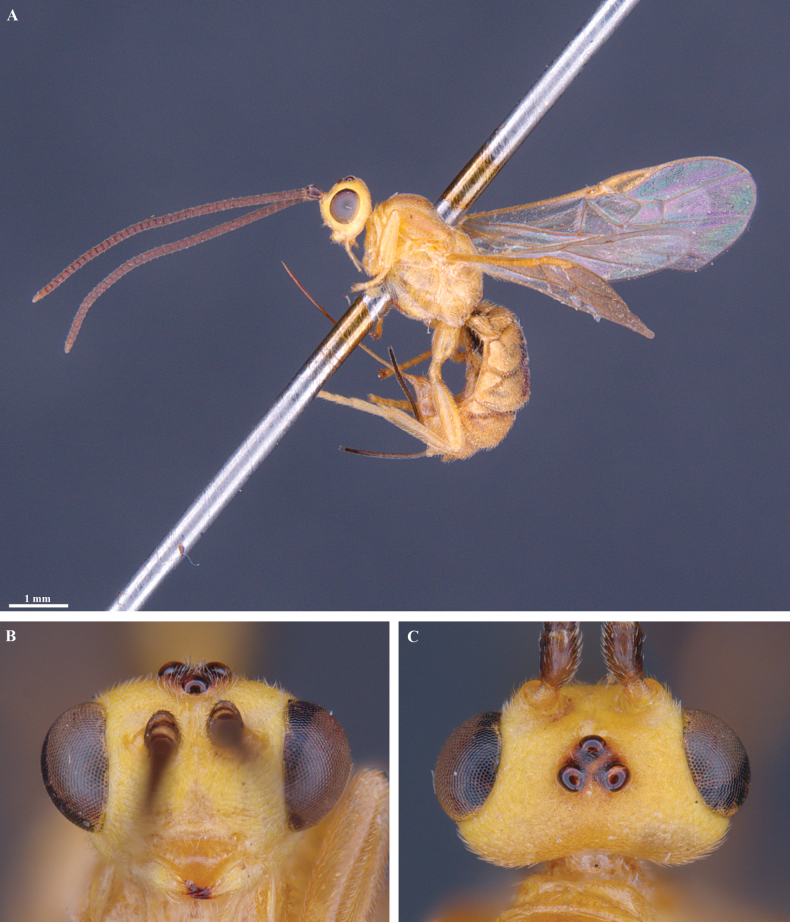
*Trigastrothecafreidbergi* sp. nov. ♀ holotype **A** habitus, lateral view **B** head, anterior view **C** head, dorsal view.

**Figure 15.**
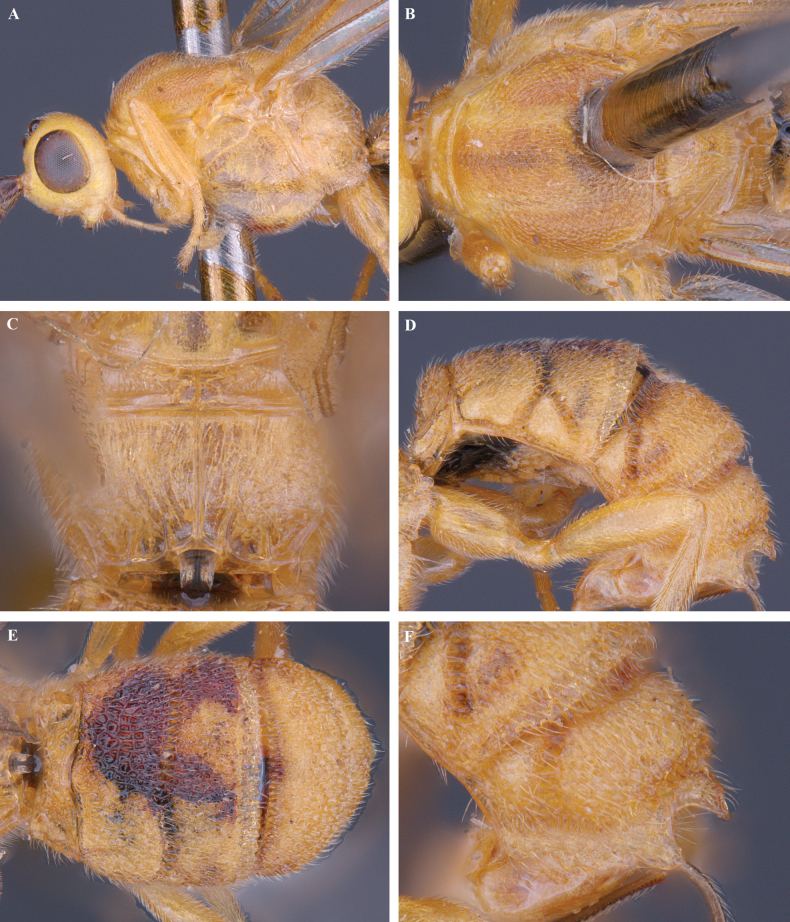
*Trigastrothecafreidbergi* sp. nov. ♀ holotype **A** head and mesosoma, lateral view **B** mesosoma, dorsal view **C** metanotum and propodeum, dorsal view **D** metasoma, lateral view **E** metasoma, dorsal view **F** T5, lateral view.

**Figure 16.**
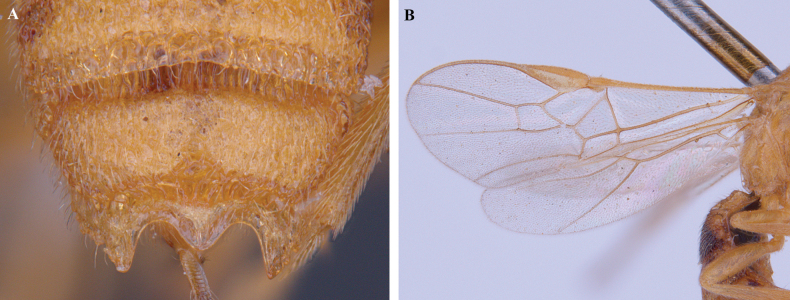
*Trigastrothecafreidbergi* sp. nov. ♀ holotype **A** T5, dorsal view **B** wings.

**Male.** Unknown.

##### Distribution.

Oriental (India).

##### Host.

Unknown.

##### Etymology.

Named after the late Dr Amnon Freidberg of Tel Aviv University, collector and prolific dipterist.

#### ﻿ Trigastrotheca griffini

Taxon classificationAnimaliaHymenopteraBraconidae

Quicke sp. nov.

8249E031-8C7B-5D9C-8CE1-A587F94743DC

https://zoobank.org/2011896B-33F1-4DAC-80FE-7EF3663901D9

Figs 17 , 18

##### Type material.

***Holotype*** ♂, Australia, New South Wales, Hat Head, 22.ii.2019, 31.0626°S, 153.052°E, 36.58 m, coll. P.D.N. Hebert, U.V. light sheet, DNA voucher P.I.D. NSWHP2575-19 (CNCO).

##### Diagnosis.

Tricolourous, largely orange-red with cream and piceous/black markings. Black mark on stemmaticum extending to form a point on vertex. Mesoscutum, orange-red except for posterior 1/2 of middle lobe which is cream, and bordered postero-laterally by piceous markings.

##### Description.

Length of body 5.5 mm, fore wing 4.1 mm. ***Head*.** Antenna with 44 flagellomeres. Terminal flagellomere acuminate. First flagellomere 1.0 × longer than 2^nd^ and 3^rd^, the latter 1.3 × longer than wide. Width of head: width of face: height of eye = 2.3: 1.1: 1.0. Face granulate with interrupted weak mid-longitudinal ridge. Inter-tentorial distance 1.4 × longer than tentorio-ocular distance. Malar suture impressed. Malar space 1.6 × as long as basal width of mandible. Antennal sockets strongly produced. Frons strongly impressed medially and behind antennal sockets with mid-longitudinal carina. Frons, vertex, and occiput granulate. Shortest distance between posterior ocelli: transverse diameter of posterior ocellus: shortest distance between posterior ocellus and eye = 1.0: 1.2: 2.2. ***Mesosoma*** 1.5 × longer than high. Mesoscutum smooth, granulate medio-posteriorly, sparsely punctate; notauli impressed anterior 1/2, crenulated, shallow posteriorly. Scutellar sulcus shallow, narrow, finely crenulate. Scutellum smooth, sparsely setose without small pit medially behind sulcus. Median area of metanotum with complete mid-longitudinal carina. Propodeum largely smooth and shiny, coarsely rugose posteriorly, distinctly transversely striate near mid-longitudinal carina; mid-longitudinal carina complete, lamelliform, bordered narrowly by fine crenulations; posteriorly propodeum with short longitudinal carinae associated with longitudinal wrinkles. ***Wings*.** Fore wing. Lengths of fore wing veins r-rs: 3RSa: 3RSb = 1.0: 1.6: 4.7. Lengths of vein 2RS: 3RSa: rs-m = 1.3: 1.3: 1.0. Base of hind wing with large glabrous area distal to vein 1CU. ***Legs*.** Lengths of fore femur: fore tibia: fore tarsus = 1.0: 1.1: 1.1. Lengths of hind femur: hind tibia: hind tarsus = 1.0: 1.1: 1.3. Claws with small acutely pointed basal lobe. ***Metasoma*.** T1 coriaceous, 1.8 × wider than long, with pair of posteriorly uniting dorsal carina. T2 1.1 × as long as T3. TT1–5 with coarse reticulate sculpture. Second metasomal suture and basal grooves of TT4 and 5 deep, strigose. T5 with postero-lateral margin convex, without emarginations. ***Coloration*.** Body mostly black except face laterally, malar space, mandible except apically, frons, and vertex laterally, T1 except medially, T2 except medially, TT3–5 antero-laterally ivory white, maxillary and labial palps, pronotum, mesoscutum, scutellum, mesopleuron, metanotum except anteriorly yellow.

**Figure 17.**
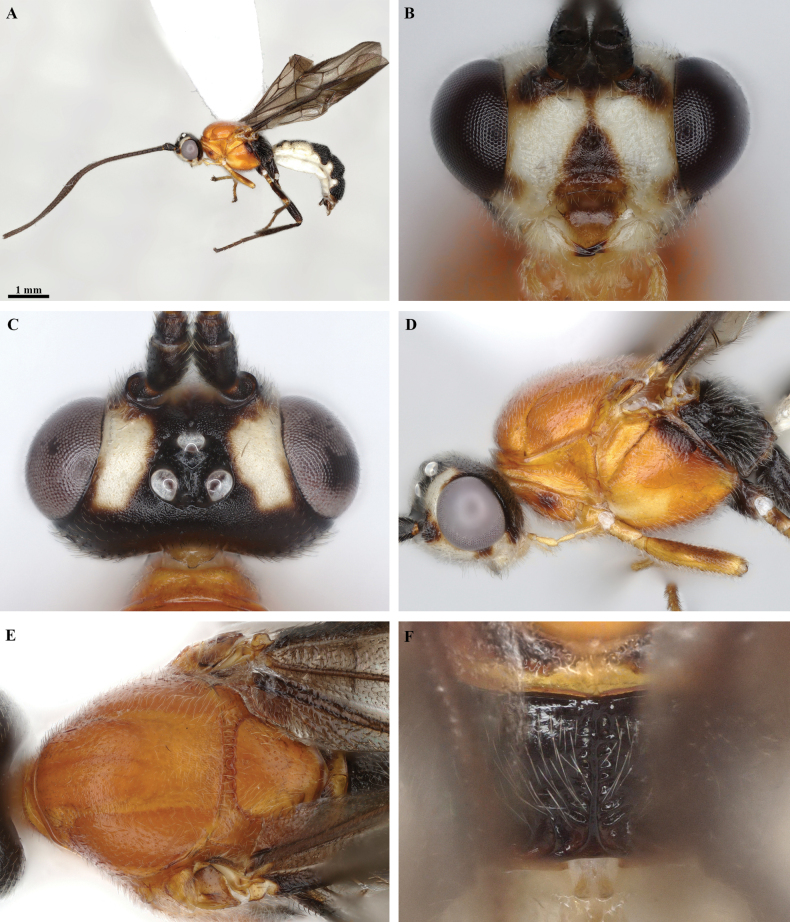
*Trigastrothecagriffini* sp. nov. ♂ holotype **A** habitus, lateral view **B** head, anterior view **C** head, dorsal view **D** head and mesosoma, lateral view **E** mesosoma, dorsal view **F** metanotum and propodeum, dorsal view.

**Figure 18.**
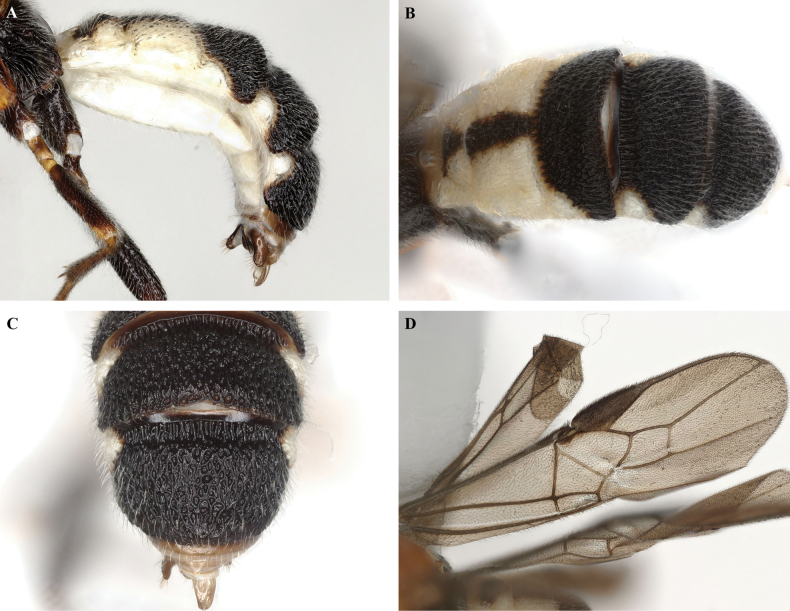
*Trigastrothecagriffini* sp. nov. ♂ holotype **A** metasoma, lateral view **B** metasoma, dorsal view **C** TT4 and 5, dorsal view **D** fore wing.

**Female.** Unknown.

##### Distribution.

Australian (Australia).

##### Host.

Unknown.

##### Etymology.

Named after Griffin Hebert who provided much assistance in the deployment of Australian Malaise traps during the fieldwork that resulted in collection of the holotype.

#### ﻿ Trigastrotheca khaoyaiensis

Taxon classificationAnimaliaHymenopteraBraconidae

Quicke & Butcher sp. nov.

E1110DC1-5151-5A24-8D24-D4601E26717F

https://zoobank.org/D3EE1FC5-DA9C-49B9-B3F8-EAC8D29B0598

Figs 19 , 20

##### Type material.

***Holotype*** ♀, Thailand, Nakhon Ratchasima, Khao Yai National Park, 17.vi.2022, 14°26.016'N, 101°22.153'E, Malaise trap 3, coll. B. Butcher (CUMZ). ***Paratype*.** 1 ♀, same data as holotype except 27.iv.2022 (CUMZ).

##### Diagnosis.

Tricolourous black, yellow, and white, with the face entirely yellow except for dark spot on outer margin of antennal socket. Similar to *T.khaoyaiensis* sp. nov. but differing in having the median lobe of T5 far more acute (Fig. 20B cf. Fig. 22D), the black raised median part of the T5 being shorter and wider with the posterior white margin relatively larger.

**Figure 19.**
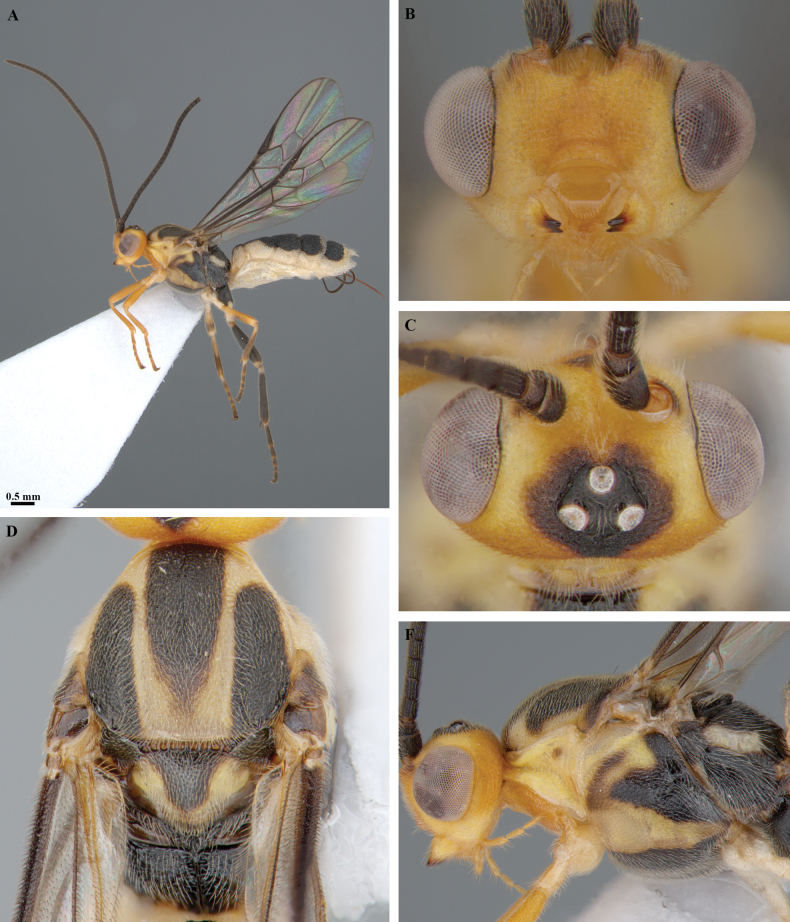
*Trigastrothecakhaoyaiensis* sp. nov. ♀ holotype **A** habitus, lateral view **B** head, anterior view **C** head, dorsal view **D** mesosoma dorsal view **E** head and mesosoma, lateral view.

**Figure 20.**
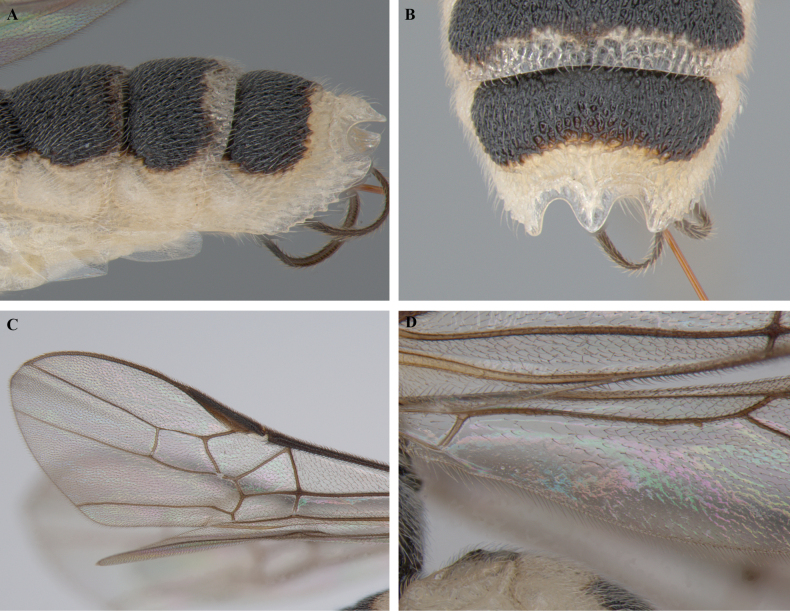
*Trigastrothecakhaoyaiensis* sp. nov. ♀ holotype **A** TT3–5, lateral view **B** TT4 and 5, dorsal view **C** fore wing **D** hind wing, basal part.

##### Description.

Holotype female. Length of body 6.1 mm, fore wing 4.5 mm. ***Head*.** Antenna with 44 flagellomeres. Terminal flagellomere acuminate. First flagellomere 1.1 × longer than 2^nd^ and 3^rd^, the latter 1.2 × longer than wide. Width of head: width of face: height of eye = 2.4: 1.3: 1.0. Face transversely striate-rugose; with distinct mid-longitudinal ridge. Inter-tentorial distance 1.6 × longer than tentorio-ocular distance. Malar suture impressed. Malar space 1.5 × as long as basal width of mandible. Antennal sockets strongly produced. Frons faintly rugose, strongly impressed behind antennal sockets, with mid-longitudinal carina. Shortest distance between posterior ocelli: transverse diameter of posterior ocellus: shortest distance between posterior ocellus and eye = 1.0: 1.0: 2.2. ***Mesosoma*** 1.5 × longer than high. Mesoscutum granulate-rugose; notauli not impressed except very short anterior part with few weak crenulations, very shallow posteriorly. Scutellar sulcus shallow, narrow, finely crenulate. Scutellum granulate without small pit medially behind sulcus. Median area of metanotum with complete mid-longitudinal carina. Propodeum granulate; mid-longitudinal carina complete, lamelliform, bordered narrowly by fine crenulations; posteriorly propodeum with short longitudinal carinae associated with longitudinal wrinkles. ***Wings*.** Fore wing. Lengths of fore wing veins r-rs: 3RSa: 3RSb = 1.0: 1.5: 4.5. Lengths of vein 2RS: 3RSa: rs-m = 1.2: 1.5: 1.0. Base of hind wing glabrous. ***Legs*.** Lengths of fore femur: fore tibia: fore tarsus = 1.0: 1.1: 1.1. Lengths of hind femur: hind tibia: hind tarsus = 1.0: 1.2: 1.3. Claws with small acutely pointed basal lobe. ***Metasoma*.** T1 1.5 × wider than long. T2 0.9 × as long as T3. T1 coriaceous. TT1–5 with coarse reticulate sculpture. Second metasomal suture and basal grooves of TT4 and 5 deep, strigose. T5 with postero-lateral margin of convex, distinctly, evenly denticulate; medial protuberance acutely rounded posteriorly; postero-lateral emarginations defined. ***Coloration*.** Body black except face, clypeus, malar space, mandible except apex, maxillary and labial palps, temple, frons and vertex laterally, occiput ventrally, pronotum, propleuron, mesoscutum antero-laterally, submedially and posteriorly, scutellum laterally and posteriorly, mesopleuron anteriorly and posteriorly, metapleuron anteriorly, fore leg, mid coxa ventrally, mid femur except ventrally, mid tibia basally, hind trochanter, hind tibia basally yellow, T1 except medially, T2 except medially and posteriorly, metasoma laterally, T5 posterior 1/2 ivory white.

**Male.** Unknown.

##### Distribution.

Oriental (Thailand).

##### Host.

Unknown.

##### Etymology.

Named after Khao Yai National Park Thailand where the type material was collected.

#### ﻿ Trigastrotheca naniensis

Taxon classificationAnimaliaHymenopteraBraconidae

Quicke & Butcher sp. nov.

EC196CDD-4307-51C4-AF57-F72F8D556086

https://zoobank.org/12F783D1-0387-4A82-AB52-111D0110B99B

Figs 21 , 22

##### Type material.

***Holotype*** ♀, Thailand, Nan Province, Doi Phu Kha National Park, 16.xii.2022, 19°12.157'N, 101°04.388'E, 1327 m, Global Malaise Trap project trap 2, coll. Butcher, B.A. (DNA voucher CCDB47579-E11) (CUMZ).

##### Diagnosis.

Tricolourous, black, yellow, ivory white, with the face entirely yellow except for dark patch on outer side of antennal socket. Similar to *T.khaoyaiensis* sp. nov. in having a yellow face without black marks but differs in having T4 completely black all the way to posterior margin.

##### Description.

Holotype female. Length of body 4.5 mm, fore wing 3.9 mm. ***Head*.** Antenna with 37 flagellomeres. Terminal flagellomere, short, sub-triangular, acuminate. First flagellomere 1.0 × longer than 2^nd^ and 3^rd^, the latter 1.5 × longer than wide. Width of head: width of face: height of eye = 2.3: 1.2: 1.0. Face transversely striate-rugose with weak mid-longitudinal ridge. Inter-tentorial distance 1.5 × longer than tentorio-ocular distance. Malar suture impressed. Malar space 1.3 × as long as basal width of mandible. Antennal sockets strongly produced. Frons strongly impressed behind antennal socket with mid-longitudinal carina. Shortest distance between posterior ocelli: transverse diameter of posterior ocellus: shortest distance between posterior ocellus and eye = 1.2: 1.0: 2.3. ***Mesosoma*** 1.5 × longer than high. Mesoscutum rugulose; notauli not impressed except very short anterior part with few weak crenulations. Scutellar sulcus shallow, narrow, finely crenulate. Scutellum faintly granulate without small pit medially behind sulcus. Median area of metanotum with complete mid-longitudinal carina. Propodeum rugose; mid-longitudinal carina complete, lamelliform, bordered narrowly by fine crenulations; posteriorly propodeum with short longitudinal carinae associated with longitudinal wrinkles. ***Wings*.** Fore wing. Lengths of fore wing veins r-rs: 3RSa: 3RSb = 1.0: 1.4: 5.3. Lengths of vein 2RS: 3RSa: rs-m = 1.2: 1.5: 1.0. Base of hind wing with large glabrous area. ***Legs*.** Lengths of fore femur: fore tibia: fore tarsus = 1.0: 1.0: 1.2. Lengths of hind femur: hind tibia: hind tarsus = 1.0: 1.3: 1.2. Claws with small acutely pointed basal lobe. ***Metasoma*.** T1 1.3 × wider than long. T2 1.1 × as long as T3. T1 coriaceous. TT1–5 with coarse reticulate sculpture. Second metasomal suture and basal grooves of TT4 and 5 deep, strigose. T5 with postero-lateral margin of convex, evenly denticulate; medial protuberance broadly rounded posteriorly. ***Coloration*.** Body black except face, clypeus, malar space, mandible except apex, maxillary and labial palps, temple, frons, and vertex laterally, occiput except medially, pronotum, propleuron, mesoscutum antero-laterally, submedially, and posteriorly, scutellum laterally and posteriorly, mesopleuron anteriorly and posteriorly, fore leg yellow, T1 except medially, T2 except medially and posteriorly, metasoma laterally, T5 posteriorly ivory white.

**Figure 21.**
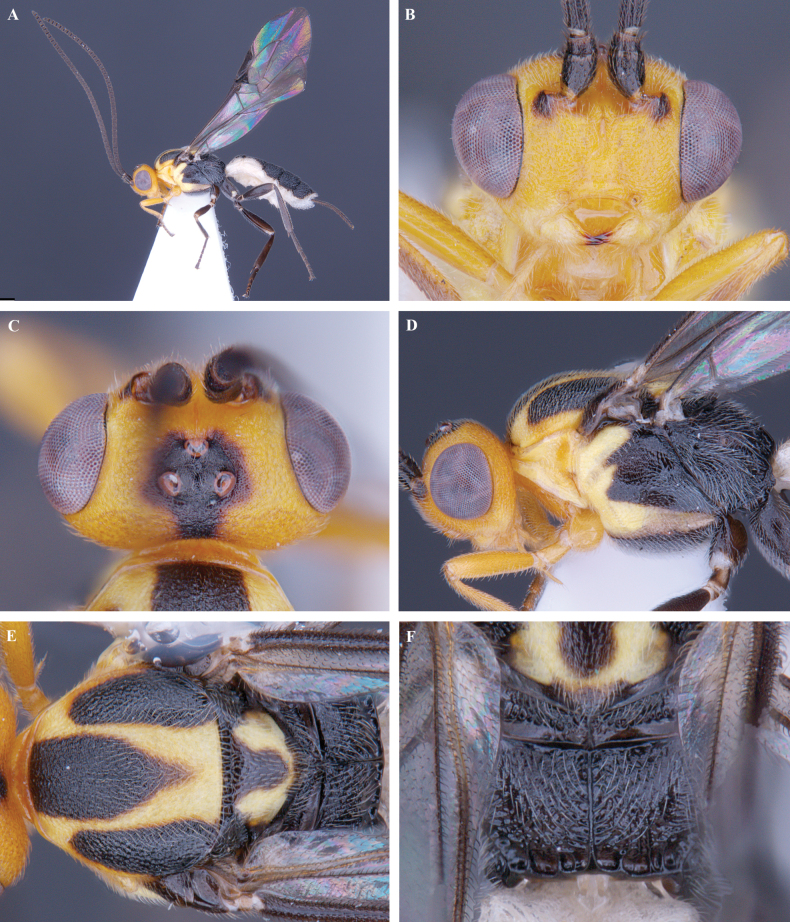
*Trigastrothecananiensis* sp. nov. ♀ holotype **A** habitus, lateral view **B** head, anterior view **C** head, dorsal view **D** head and mesosoma, lateral view **E** mesosoma, dorsal view **F** metanotum and propodeum, dorsal view.

**Figure 22.**
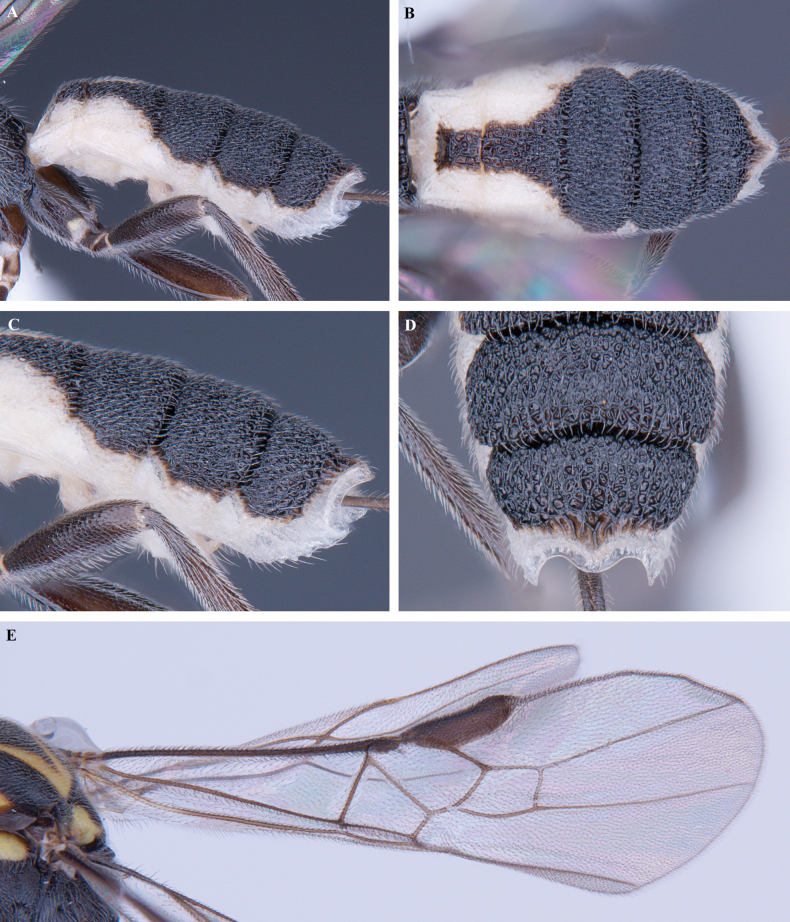
*Trigastrothecananiensis* sp. nov. ♀ holotype **A** metasoma, lateral view **B** metasoma, dorsal view **C** TT3–5, lateral view **D** TT4 and 5, dorsal view **E** wings.

**Male.** Unknown.

##### Distribution.

Oriental (Thailand).

##### Host.

Unknown.

##### Etymology.

Named after the province of Nan in north Thailand where the holotype was collected.

#### ﻿ Trigastrotheca simba

Taxon classificationAnimaliaHymenopteraBraconidae

van Noort sp. nov.

44EE9E84-E1DB-5B44-83BB-7060D2160B0B

https://zoobank.org/C0B3DA61-2120-4065-AA70-18618CDBD032

Figs 23 , 24

##### Type material.

***Holotype*** ♀, Tanzania, Mkomazi Game Reserve, Kamakota Hill, 4.14°S, 28.24°E, 4 Dec 1995, S. van Noort, on *Ficusingens* (Miq.) Miq. With ripe fig crop (SAMC). ***Paratypes***: 1 ♂, same data as holotype (SAMC).

##### Diagnosis.

Similar to *T.trilobata* but tarsal claw with a smaller, bidentate, and more rounded basal lobe compared to a larger, more foliaceous, and acutely triangular basal lobe in *T.trilobata*. The postero-lateral margin of T5 has more prominent teeth (~ 8 or 9) compared to *T.trilobata* which has only 5 or fewer obvious teeth (Fig. 33D). Postero-medial protuberance of T5 acutely rounded, shorter than lateral lobes, with concavities lateral of medial protuberance acutely invaginated, whereas in *T.trilobata* the medial protuberance is of equivalent length to the lateral lobes; and the concavities lateral of medial protuberance are broadly invaginated.

##### Description.

Holotype female. Length of body 5.16 mm, fore wing 4.14 mm. ***Head*.** Antenna with 36 flagellomeres. Terminal flagellomere with a spicule. First flagellomere equivalent in length to the 2^nd^ and 3^rd^, the latter 1.25 × longer than wide. Width of head: width of face: height of eye = 2.5: 1.3: 1.0. Face with fine transverse striations laterally; with mid-longitudinal ridge dissipating ventrally. Inter-tentorial distance 2.3 × longer than tentorio-ocular distance. Malar suture impressed. Malar space equivalent to basal width of mandible. Frons strongly impressed with a complete mid-longitudinal carina. Shortest distance between posterior ocelli: transverse diameter of posterior ocellus: shortest distance between posterior ocellus and eye = 1.0: 1.0: 2.3. ***Mesosoma*** 1.5 × longer than high. Mesoscutum rugose; notauli weakly impressed for entire length with weak crenulations. Scutellar sulcus shallow, narrow, finely crenulate. Scutellum weakly rugose, setose without small pit medially behind sulcus. Median area of metanotum with complete mid-longitudinal carina. Propodeum weakly rugose; mid-longitudinal carina complete, lamelliform, with ~ 9 transverse, curved striae radiating laterally for short distance before grading into rugulosity; posteriorly propodeum with short longitudinal carinae. ***Wings*.** Fore wing. Lengths of fore wing veins r-rs: 3RSa: 3RSb = 1.0: 1.5: 4.6. Lengths of vein 2RS: 3RSa: rs-m = 1.0: 1.3: 1.8. Base of hind wing glabrous. ***Legs*.** Lengths of fore femur: fore tibia: fore tarsus = 1.0: 1.2: 1.2. Lengths of hind femur: hind tibia: hind tarsus = 1.0: 1.1: 1.0. Claws with small rounded weakly bilobate basal lobe. ***Metasoma*.** T1 2.0 × wider than long. T2 0.7 × as long as T3. TT1–5 with coarse reticulate sculpture. Second metasomal suture weakly strigose, basal grooves of TT4 and 5 deep, strigose. T5 with postero-lateral margin convex, distinctly denticulate (~ 8 or 9 uneven teeth) along anterior 3/4 of margin; medial protuberance acutely rounded, shorter than lateral lobes, concavities lateral of medial protuberance acutely invaginated. ***Coloration*.** Body mostly yellow except antenna, eyes, and tarsi black, and stemmaticum which is dark brown with orange margins.

**Figure 23.**
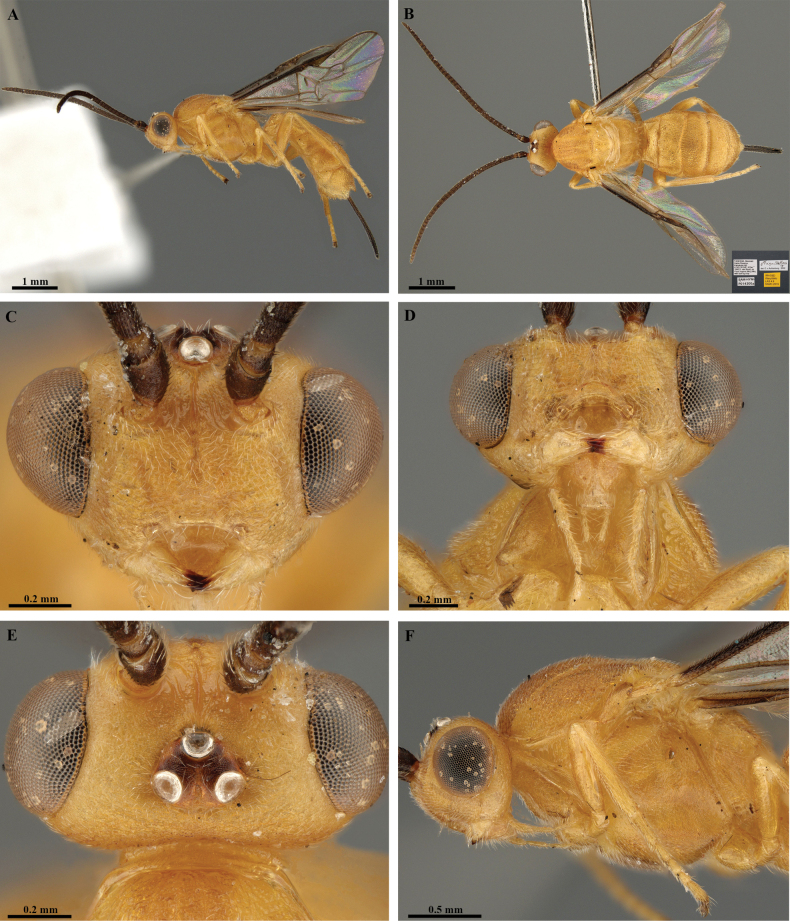
*Trigastrothecasimba* sp. nov. ♀ holotype **A** habitus, lateral view **B** habitus, dorsal view with labels inset **C** head, anterior view **D** head, antero-ventral view **E** head, dorsal view **F** head and mesosoma, lateral view.

**Figure 24.**
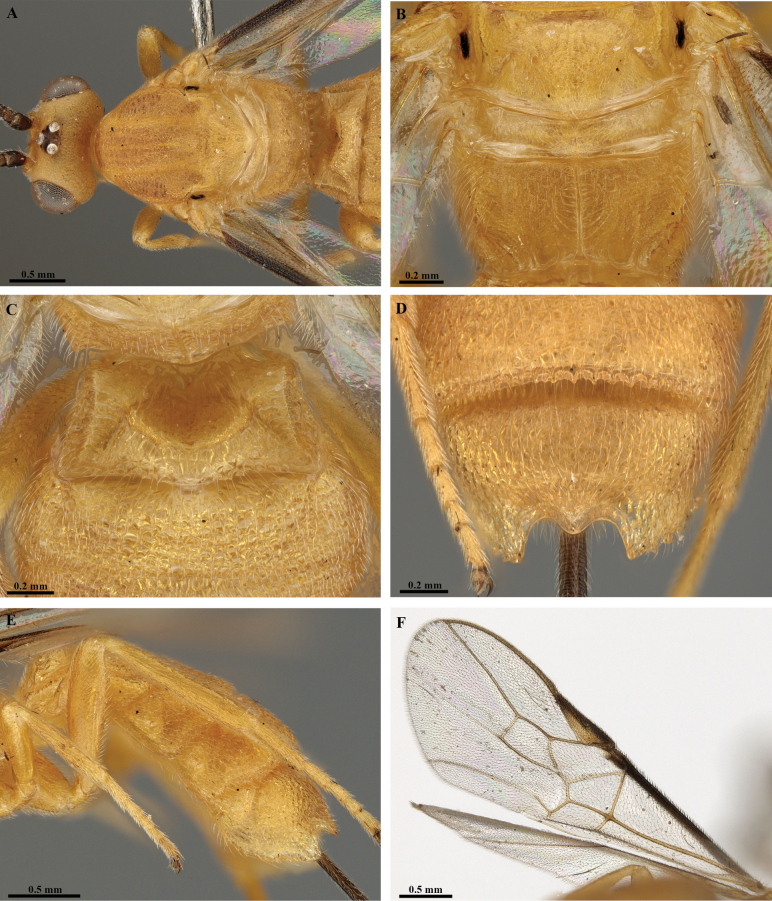
*Trigastrothecasimba* sp. nov. ♀ holotype **A** head and mesosoma, dorsal view **B** scutellum, metanotum and propodeum, dorsal view **C** T1, dorsal view **D** T5, dorsal view **E** metasoma, lateral view **F** fore wing.

**Male.** Same as female.

##### Distribution.

Afrotropical (Tanzania).

##### Host.

Unknown, but both the female and male were hand collected from a *Ficusingens* tree with a ripe fig crop, and they may have been locating or recently emerged from arboreal nesting *Crematogaster* on the tree.

##### Etymology.

Specific name refers to the presence alongside wild lions of this uniformly pale colored species in the East African savanna (Mkomazi Game Reserve, now a National Park), the provenance of the holotype. Simba is Kiswahili for lion. Noun in apposition.

#### ﻿ Trigastrotheca similidentatata

Taxon classificationAnimaliaHymenopteraBraconidae

Ranjith sp. nov.

D6301B56-52B3-549C-B719-1490FA4CA09C

https://zoobank.org/0FF021B2-B86C-40A7-8103-0977CD70B1F4

Figs 25 , 26

##### Type material.

***Holotype*** ♀, India: Karnataka, Chamarajanagar, Biligiri Rangaswamy Temple Tiger Reserve, scrub jungle, 12°01'41.4"N, 77°06'55.1"E; 31.v–15.vi.2005, Malaise trap, coll. D.R. Priyadarsanan (AIMB). ***Paratype***, 1 ♀, India: Karnataka, Kadnur, Malaise Trap, 14.xi.2005, coll. D.R. Priyadarsanan (DZUC).

##### Diagnosis.

Similar to *T.tridentata* in having an entirely black mesoscutum except for yellow notauli in having the medio-posterior lobe of T5 broadly rounded (Fig. 26D) (far more acutely pointed in *T.tridentata*) and in the posterolateral parts of the T5 not concave laterally. In addition, the black mark at the base of T5 does not extend to the lateral margins whereas in *T.tridentata* the black is produced reaching the side and posterior margins.

**Figure 25.**
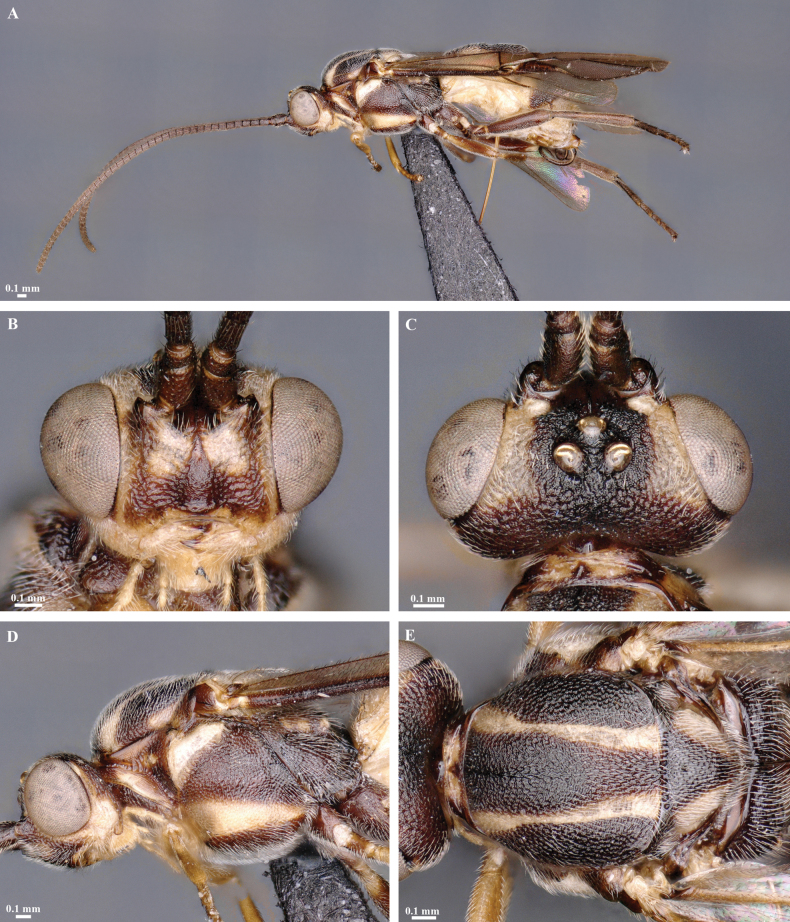
*Trigastrothecasimilidentata* sp. nov. ♀ holotype **A** habitus, lateral view **B** head, anterior view **C** head, dorsal view **D** head and mesosoma, lateral view **E** mesosoma, dorsal view.

**Figure 26.**
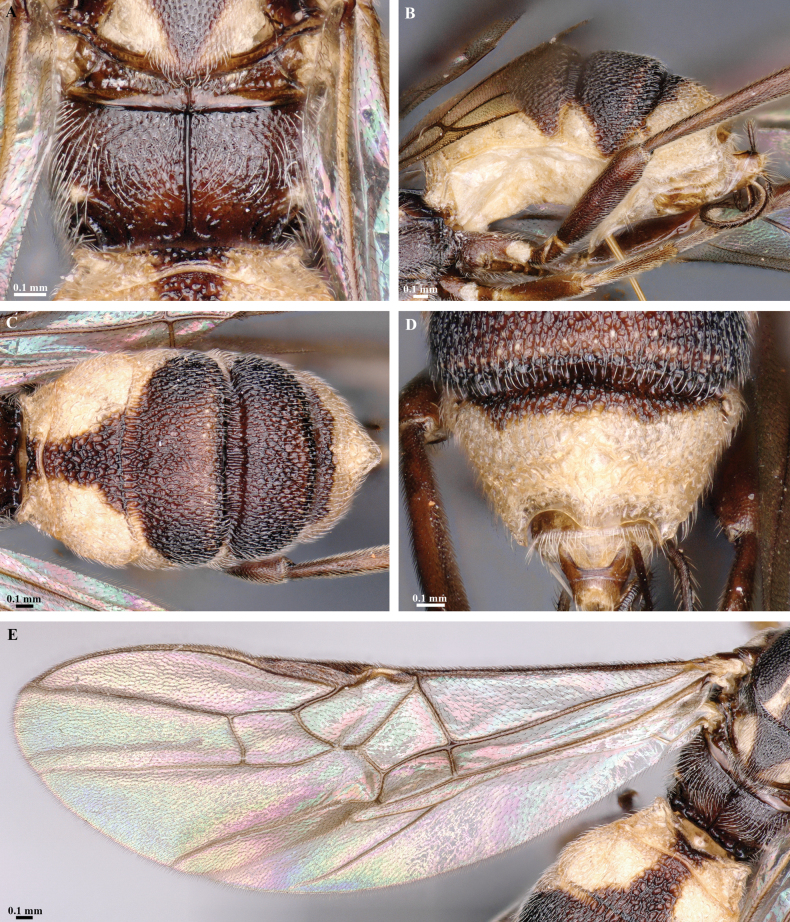
*Trigastrothecasimilidentata* sp. nov. ♀ holotype **A** metanotum and propodeum, dorsal view **B** metasoma, lateral view **C** metasoma, dorsal view **D** T5, dorsal view **E** wings.

##### Description.

Length of body 3.6 mm, fore wing 3.1 mm. ***Head*.** Antenna incomplete with 42 flagellomeres. Terminal flagellomere acuminate. First flagellomere 1.1 × longer than 2^nd^ and 3^rd^, the latter 1.3 × longer than wide. Width of head: width of face: height of eye = 2.2: 1.1: 1.0. Face transversely striate-rugose with long mid-longitudinal ridge. Inter-tentorial distance 1.8 × longer than tentorio-ocular distance. Malar suture impressed. Malar space 1.5 × as long as basal width of mandible. Antennal sockets strongly produced. Frons strongly impressed only behind antennal sockets with mid-longitudinal carina. Frons, vertex, and occiput granulate-rugose. Shortest distance between posterior ocelli: transverse diameter of posterior ocellus: shortest distance between posterior ocellus and eye = 1.0: 1.4: 2.4. ***Mesosoma*** 1.5 × longer than high. Mesoscutum transversely rugose-striate with a weak mid-longitudinal carina medio-anteriorly; notauli impressed anterior 1/2, crenulated, shallow posteriorly. Scutellar sulcus deep, narrow, finely crenulate. Scutellum distinctly punctate, setose with a small pit medially behind sulcus. Median area of metanotum with complete mid-longitudinal carina. Propodeum faintly rugose, mid-longitudinal carina complete, lamelliform posteriorly, bordered narrowly by fine crenulations; posteriorly propodeum with short longitudinal carinae associated with longitudinal wrinkles. ***Wings*.** Fore wing. Lengths of fore wing veins r-rs: 3RSa: 3RSb = 1.0: 1.1: 3.7. Lengths of vein 2RS: 3RSa: rs-m = 1.0: 1.1: 1.0. Base of hind wing with large glabrous area distal to vein 1CU. ***Legs*.** Lengths of fore femur: fore tibia: fore tarsus = 1.0: 1.2: 1.2. Lengths of hind femur: hind tibia: hind tarsus = 1.0: 1.2: 1.4. Claws with small acutely pointed basal lobe. ***Metasoma*.** T1 coarsely reticulate, 1.7 × wider than long, with pair of posteriorly uniting dorsal carina. T2 0.7 × as long as T3. TT1–5 with coarse reticulate sculpture. Second metasomal suture and basal grooves of TT4 and 5 deep, strigose. T5 with postero-lateral margin slightly concave, denticulate; medial protuberance broadly rounded posteriorly; postero-lateral emarginations concave. ***Coloration*.** Body mostly reddish brown except scape, pedicel, eye, ocellar area, occiput medially, mesoscutum antero-laterally, propleuron, mesopleuron with posterior black patch, T1 medially, ovipositor sheath black, face, clypeus, maxillary and labial palps, frons, vertex, and occiput laterally, pronotum dorsally, legs yellow, pronotum laterally, middle lobe of mesoscutum posteriorly, mesopleuron anteriorly, metasoma laterally ivory white.

**Male.** Unknown.

##### Distribution.

Oriental (India).

##### Host.

Unknown.

##### Etymology.

From Latin, *similis* and *dentata*, in reference to its likeness to *T.tridentata*.

#### ﻿ Trigastrotheca sublobata

Taxon classificationAnimaliaHymenopteraBraconidae

Quicke sp. nov.

29AC84E0-C56E-5D39-B319-9AA6E74A8E4F

https://zoobank.org/3C2251FF-3194-47AB-9CC1-34407BFE2E0E

Fig. 27

##### Type material.

***Holotype*** ♀, Thailand: Nan Province, Phasing, 2018–2019, light trap, coll. Chansri, K. (CUMZ).

##### Diagnosis.

**Figure 27.**
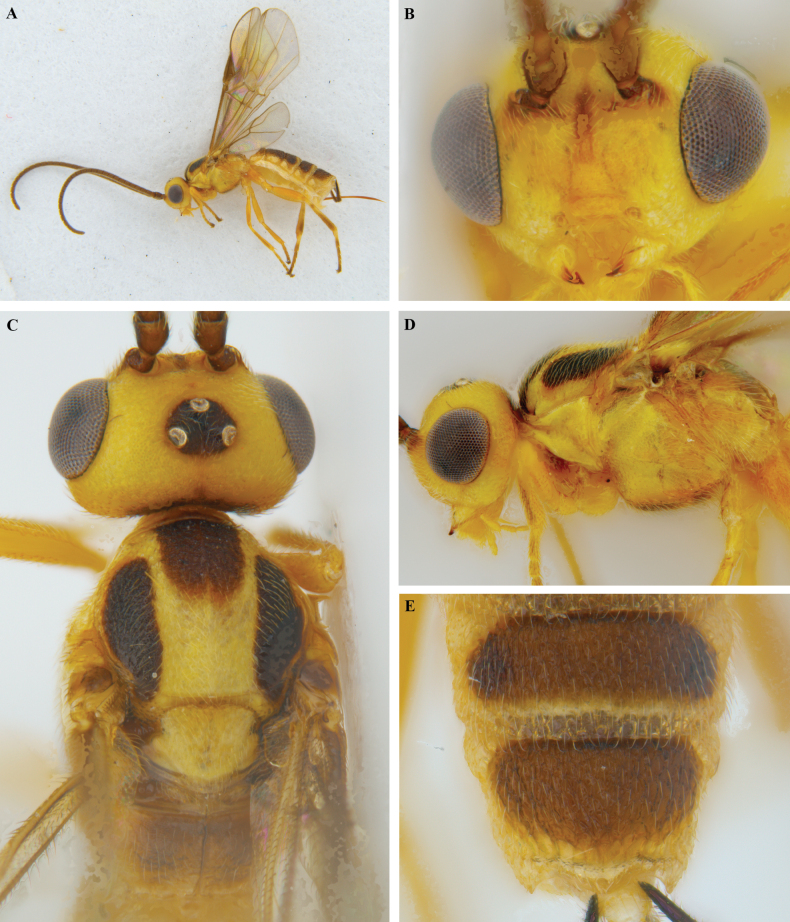
*Trigastrothecasublobata* sp. nov. ♀ holotype **A** habitus, lateral view **B** head, anterior view **C** head and mesosoma, dorsal view **D** head and mesosoma, lateral view **E** TT4 and 5, dorsal view.

May be distinguished from the other Oriental species with three black patches on the mesoscutum and medial black patches on most of the metasomal tergites by its only weakly produced and very wide middle lobe of T5 (Fig. 27E).

##### Description.

Holotype female. Length of body 3.1 mm, fore wing 2.7 mm. ***Head*.** Antenna with 28 flagellomeres. Terminal flagellomere, short, sub-triangular, acuminate. First flagellomere 1.0 × longer than 2^nd^ and 3^rd^, the latter 1.7 × longer than wide. Width of head: width of face: height of eye = 2.5: 1.3: 1.0. Face with fine transverse striations radiating towards middle from eye margin; with weak mid-longitudinal ridge. Inter-tentorial distance 1.1 × longer than tentorio-ocular distance. Malar suture impressed. Malar space 1.6 × as long as basal width of mandible. Antennal sockets strongly produced. Frons hardly impressed behind antennal socket with shallow mid-longitudinal groove. Shortest distance between posterior ocelli: transverse diameter of posterior ocellus: shortest distance between posterior ocellus and eye = 1.5: 1.0: 2.8. ***Mesosoma*** 1.5 × longer than high. Mesoscutum granulate; notauli not impressed except very short anterior part with few weak crenulations. Scutellar sulcus shallow, narrow, finely crenulate. Scutellum finely granulate without small pit medially behind sulcus. Median area of metanotum with complete mid-longitudinal carina. Propodeum granulate; mid-longitudinal carina complete, not lamelliform, not bordered narrowly by fine crenulations; posteriorly propodeum with short longitudinal carinae associated with few longitudinal wrinkles. ***Wings*.** Fore wing. Lengths of fore wing veins r-rs: 3RSa: 3RSb = 1.0: 1.0: 4.1. Lengths of vein 2RS: 3RSa: rs-m = 1.3: 1.2: 1.0. Base of hind wing distinctly setose. ***Legs*.** Lengths of fore femur: fore tibia: fore tarsus = 1.0: 1.0: 1.2. Lengths of hind femur: hind tibia: hind tarsus = 1.0: 1.3: 1.2. Claws with small acutely pointed basal lobe. ***Metasoma*.** T1 1.6 × wider than long. T2 1.1 × as long as T3. T1 coriaceous. TT1–5 with coarse reticulate sculpture. Second metasomal suture and basal grooves of TT4 and 5 deep, strigose. T5 with postero-lateral margin of convex, unevenly denticulate; medial protuberance broadly rounded posteriorly. ***Coloration*.** Body yellow except mandible apically, stemmaticum, occiput ventrally, middle lobe of mesoscutum anterior 1/2, lateral lobe of mesoscutum, tegula, propleuron posteriorly, mesopleuron ventrally, propodeum anterior 1/2, hind tibia apical 1/2, hind tarsus, T1 postero-medially, T2 except laterally, TT3–5 except laterally and posteriorly, ovipositor sheath brown.

**Male.** Unknown.

##### Distribution.

Oriental (Thailand).

##### Host.

Unknown.

##### Etymology.

Name refers to the relatively short medial and lateral lobes of the T5.

### ﻿Notes on described species

#### ﻿ Trigastrotheca acroceropsis

Taxon classificationAnimaliaHymenopteraBraconidae

Ranjith & Quicke nom. nov.

15FC0529-909C-5D51-BB69-B0B5126CC072

Figs 27 , 28

Acrocerilia tricolor Quicke & Ingram, 1993: 302, 306.

##### Diagnosis.

**Figure 28.**
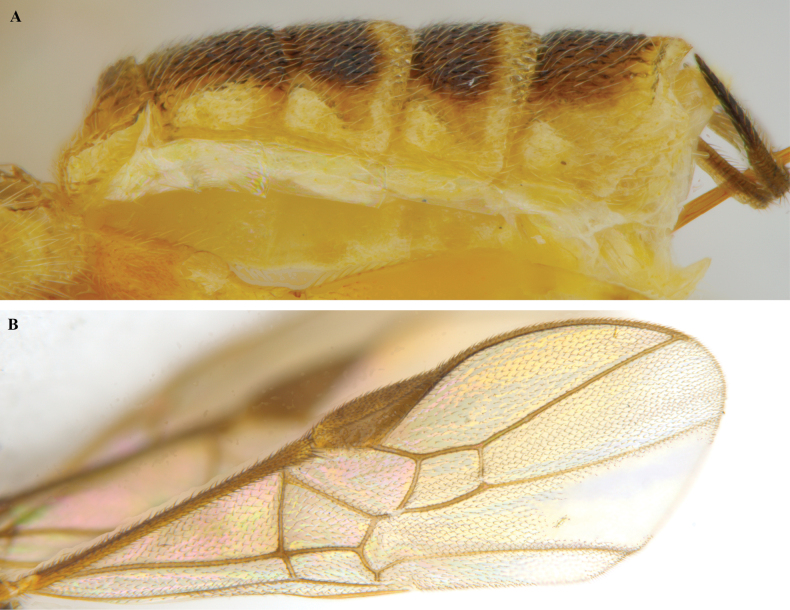
*Trigastrothecasublobata* sp. nov. ♀ holotype **A** metasoma, lateral view **B** fore wing.

Tricolourous, orange-red, black, and ivory white. Mesoscutum orange-red without dark marks as in *T.griffini* Quicke, sp. nov. but differs in the black central area of T2 being far larger and widening posteriorly. Basal lobe of claw largely rounded with small distal tooth (Fig. 28D). In addition, see Quicke and Ingram (1993: 304, 306).

##### Notes.

After re-examining images of the male holotype *Acroceriliatricolor*, we found that it is not conspecific with the type species of *Acrocerilia*, viz. *A.pachynervis* van Achterberg, 1989, also based on a male specimen, and we hereby formally transfer it to *Trigastrotheca*. The species name *tricolor* is preoccupied in the genus *Trigastrotheca* (*T.tricolor* Quicke & Ingram, 1993). *Trigastrothecatricolor* is therefore a secondary homonym. Here we propose *T.acroceropsis* Ranjith & Quicke as a replacement name.

#### ﻿ Trigastrotheca nigricornis

Taxon classificationAnimaliaHymenopteraBraconidae

Cameron, [1909] 1910

35397D1E-8EE9-50C5-B95B-4E83BC7EFCF6

Figs 29 , 30

##### Notes.

The holotype female from South Africa (Cape Colony) is in the MFN but is too badly eaten by dermestids (Shenefelt 1978) to permit species-level identification, and it is therefore not included in the key. However, the original description is fairly complete for the time and includes the following diagnosis for distinguishing it from *T.trilobata*: “May be known from the type species of the genus, *T.trilobata*, Cam. (Ann. S. Afr. Mus., V, 32), by the shorter ovipositor (4 mm with a body length of 5 mm in the latter), by the middle lobe on the apex of the abdomen being hardly developed and by the 1^st^ abscissa of the radius being longer, not shorter, than the 2^nd^.”

**Figure 29.**
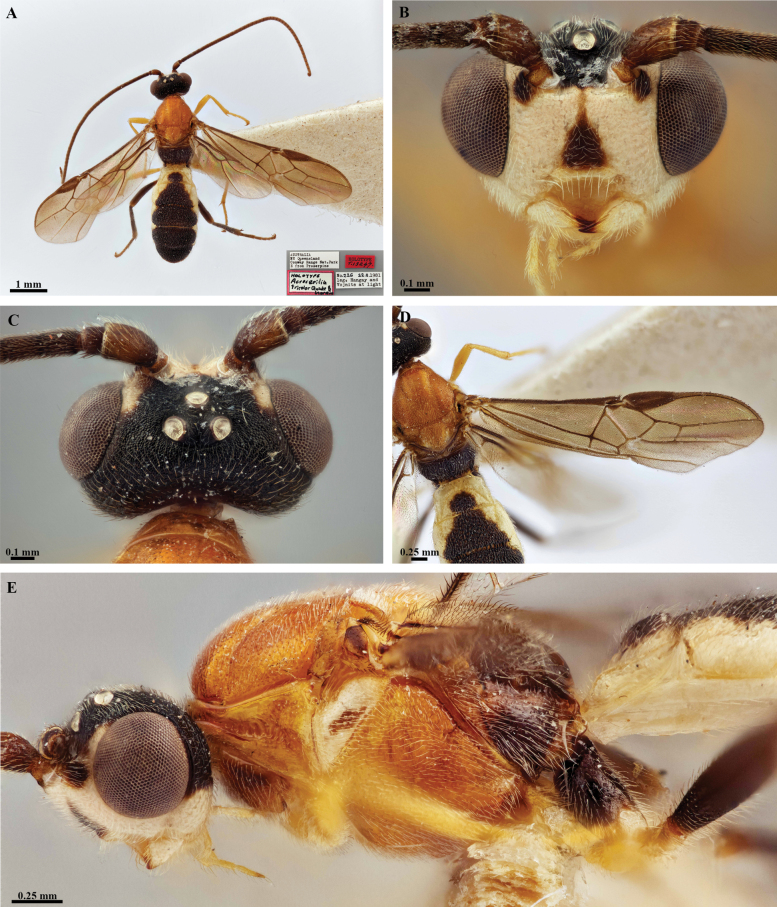
*Trigastrothecaacroceropsis* nom. nov. ♂ holotype **A** habitus, dorsal view with labels inset **B** head, anterior view **C** head, dorsal view **D** mesosoma, anterior metasoma and wings, dorsal view **E** head and mesosoma, lateral view.

**Figure 30.**
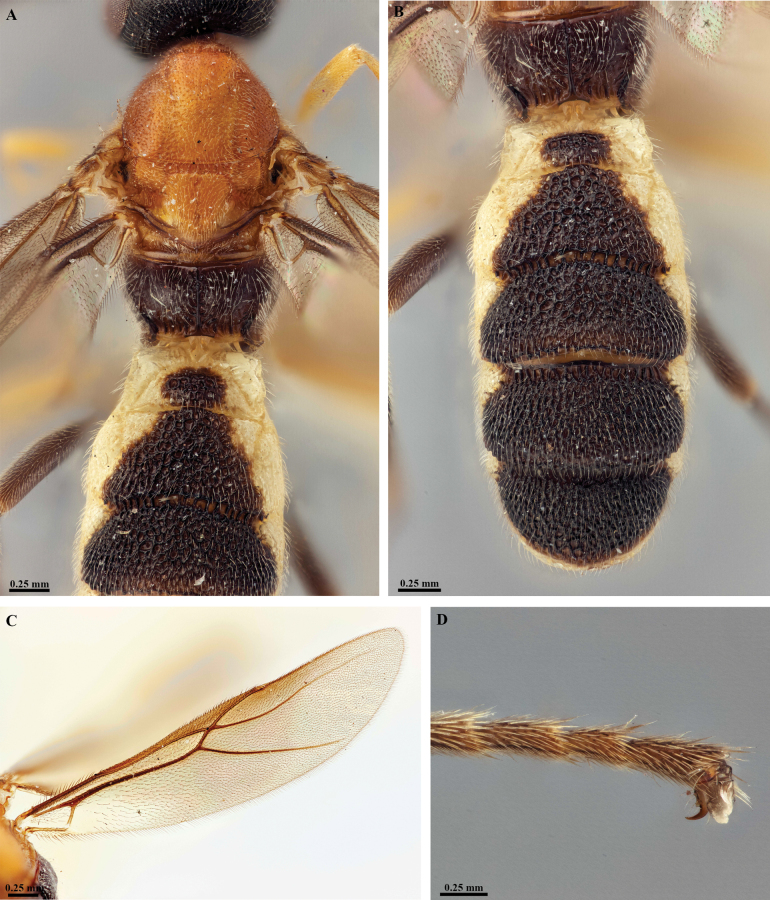
*Trigastrothecaacroceropsis* nom. nov. ♂ holotype **A** mesosoma and anterior metasoma, dorsal view **B** propodeum and metasoma, dorsal view **C** hind wing **D** distal hind tarsomeres and claw.

#### ﻿ Trigastrotheca romani

Taxon classificationAnimaliaHymenopteraBraconidae

Quicke, 2005

65E9898E-5D64-52C2-8B30-39B1F9989EBE

Fig. 31

##### Diagnosis.

Similar to *T.trilobata* but differs in the frons anterolateral to median ocellus without diverging striae, the second metasomal suture being less strongly arched medially (Fig. 33F cf. Fig. 37B) and the posteromedial lobe of T5 being less sharp and protruding.

**Figure 31.**
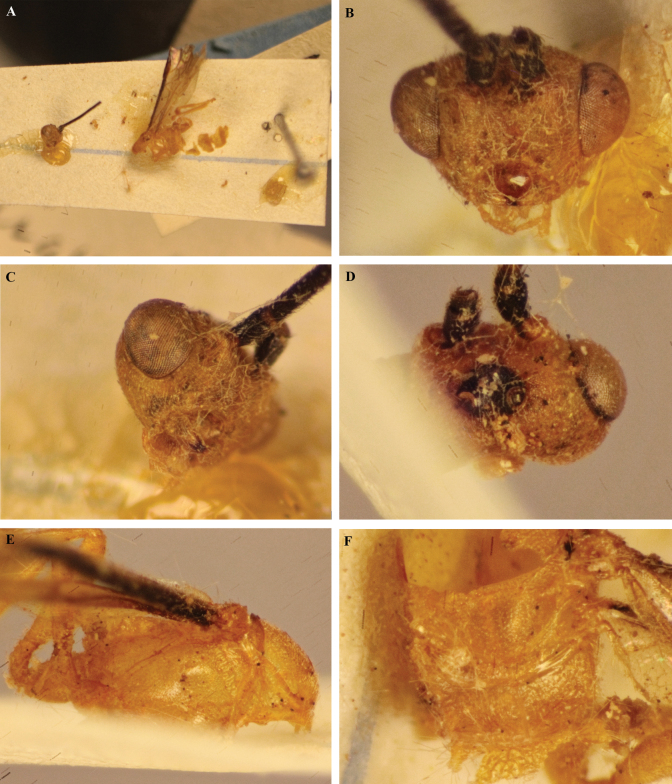
*Trigastrothecanigricornis* ♀ holotype **A** remains on card rectangle **B** head, anterior view **C** head, oblique anterior view **D** head, dorsal view **E** mesosoma, lateral view **F** scutellum and propodeum, dorsal view.

#### ﻿ Trigastrotheca sureeratae

Taxon classificationAnimaliaHymenopteraBraconidae

Quicke & Butcher, 2017

66320483-86C1-5FAD-8B26-AEB0000269A2

Figs 32 , 33

##### Material examined.

1 ♀, Thailand, Nan Province, Sakaerat, 22.iv.2022 (CUMZ). DNA voucher P.I.D. BBTH3136-22.

##### Diagnosis, female.

Similar to male except body length 5.0 mm. Antenna with 38 flagellomeres. Median flagellomeres as long as wide. First flagellomere 1.0 × longer than 2^nd^ and 3^rd^, respectively, 1.6 × longer than wide. Width of head: width of face: height of eye = 2.4: 1.3: 1.0. Inter tentorial distance: tentorio-ocular distance = 1.5: 1.0. Frons with strong mid-longitudinal carina. Shortest distance between posterior ocelli: transverse diameter of posterior ocellus: shortest distance between posterior ocellus and eye = 1.3: 1.0: 3.0. Lengths of veins r-rs: 3RSa: 3RSb = 1.0: 1.3: 4.6. Lengths of veins 2RS: 3RSa: rs-m = 1.2: 1.2: 1.0. Base of hind wing without large glabrous area. Lengths of fore femur: fore tibia: fore tarsus = 1.0: 1.0: 1.3. Lengths of hind femur: hind tibia: hind tarsus = 1.0: 1.4: 1.3. T2 with parallel sided sublateral grooves. Second metasomal suture straight. Median length of T3 1.1 × T2. Mesoscutum with three longitudinal black patches. Propodeum black anterior 2/3. T2 with narrow black longitudinal patch.

**Figure 32.**
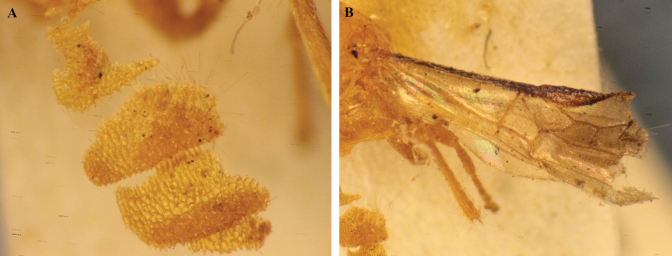
*Trigastrothecanigricornis* ♀ holotype **A** remains of tergites, dorsal view **B** remains of wings.

**Figure 33.**
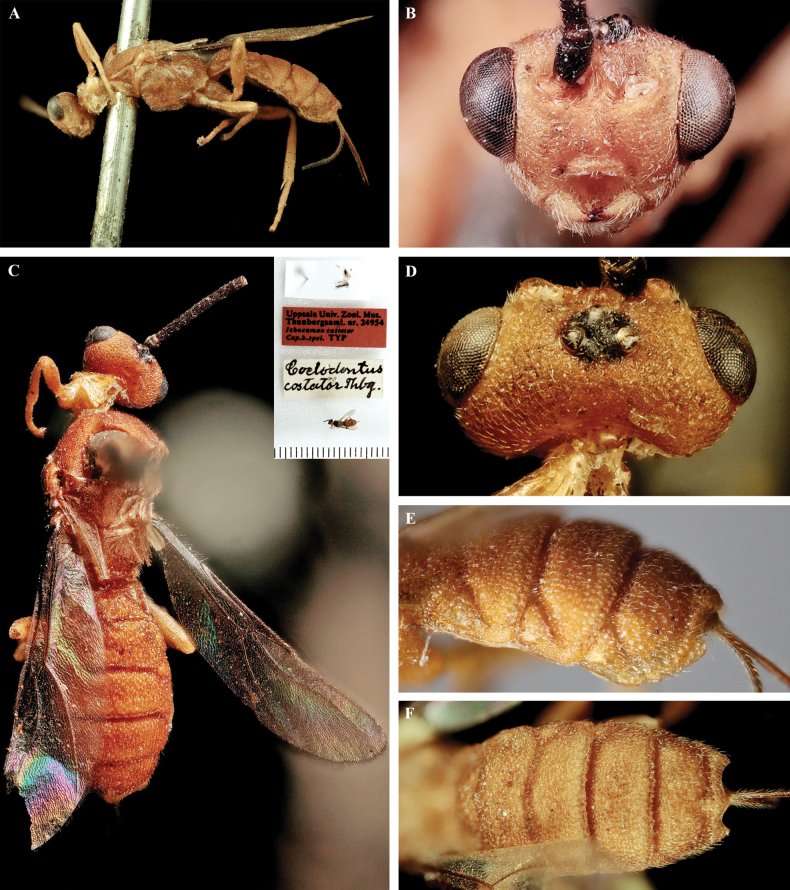
*Trigastrothecaromani* ♀ holotype **A** habitus, lateral view **B** head, anterior view **C** habitus dorsal view [notes: color is artefactually redder than in reality; labels inset] **D** head, dorsal view **E** TT2–5, lateral view **F** metasoma, dorsal view.

##### Notes.

The female specimen of *T.sureeratae* is comes close to *T.laikipiensis* and the differences are mentioned in the taxonomic key.

#### ﻿ Trigastrotheca tricolor

Taxon classificationAnimaliaHymenopteraBraconidae

Quicke & Ingram, 1993

E646748D-E2D7-5638-A1EF-8A813A8890DD

##### Diagnosis.

The color pattern is unique in the genus, viz. largely ochraceous yellow but head piceous with pair large, sub-rectangular areas laterally on the frons; the notauli, posterior 1/2 of middle lobe of mesoscutum and scutellum paler; metasomal tergites bicolorous, ochraceous except TT1 and 2 and anterolateral corners of T3 and posterior margin of T5 which are ivory-white. In addition, see Quicke and Ingram (1993: 331–332, figs 3, 87, 112, 113).

#### ﻿ Trigastrotheca tridentata

Taxon classificationAnimaliaHymenopteraBraconidae

Enderlein, [1918] 1920

D2472CB4-A520-517C-AD0C-D94B25F759B7

##### Diagnosis.

Tricolourous, black, cream, sulphur-yellow. This is the only known species which has the postero-lateral margin of the T5 concave with an abrupt lateral angulation (see Raweearamwong et al. 2020: 183, fig. 3F).

#### ﻿ Trigastrotheca trilobata

Taxon classificationAnimaliaHymenopteraBraconidae

Cameron, 1906

9591492F-E1D0-5168-A10C-8D1065FD916F

Figs 34 , 35

##### Diagnosis.

Similar to *T.aethiopica* sp. nov. and *T.romani* in being ochraceous except for black stemmaticum. Differs from *T.aethiopica* sp. nov. in having the pterostigma largely black, especially on the anterior 1/2, and from *T.romani* in that it has the frons anterolateral to median ocellus with distinct weakly diverging striae which is similar to the condition found in the male of *T.serrata* (van Achterberg & Sigwalt, 1987).

##### Notes.

The type species of *Trigastrotheca* is *T.trilobata* Cameron, 1906 from South Africa or Zimbabwe [Rhodesia] (Cameron 1906). The type label in Cameron’s handwriting states “Cape” (Fig. 32A), but on the underside of the card that the specimen is micro-pinned to with a minute pin “Rhodesia” and an illegible word is written (see http://www.waspweb.org/ichneumonoidea/Braconidae/Braconinae/Trigastrotheca/Trigastrotheca_trilobata.htm).

**Figure 34.**
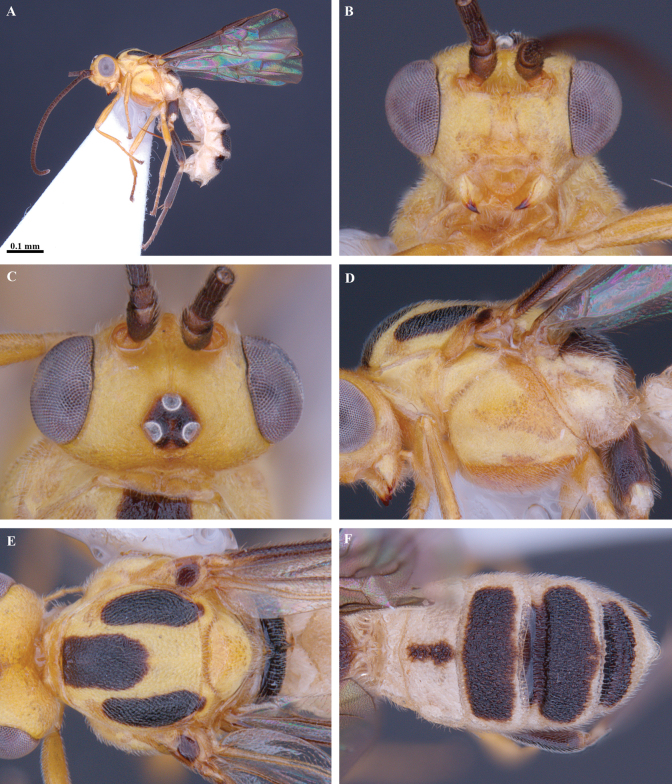
*Trigastrothecasureeratae* ♀ A. habitus, lateral view **B** head, anterior view **C** head, dorsal view **D** mesosoma, lateral view **E** mesosoma, dorsal view **F** metasoma, dorsal view.

**Figure 35.**
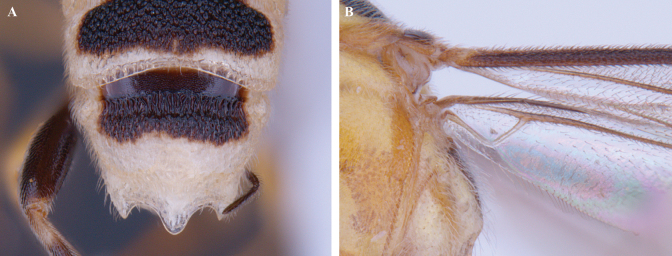
*Trigastrothecasureeratae* ♀ **A** T5, dorsal view **B** base of hind wing

**Figure 36.**
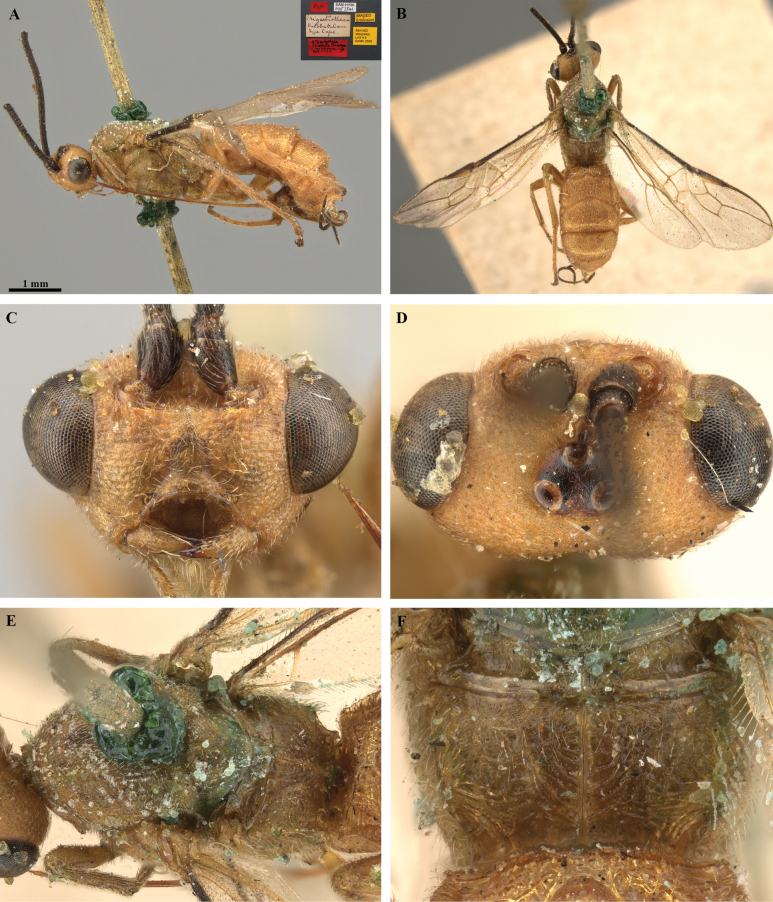
*Trigastrothecatrilobata* ♀ holotype **A** habitus, lateral view with labels inset **B** habitus, dorsal view **C** head, anterior view **D** head, dorsal view, E, mesosoma, slightly oblique dorsal view **F** metanotum and propodeum, dorsal view.

**Figure 37.**
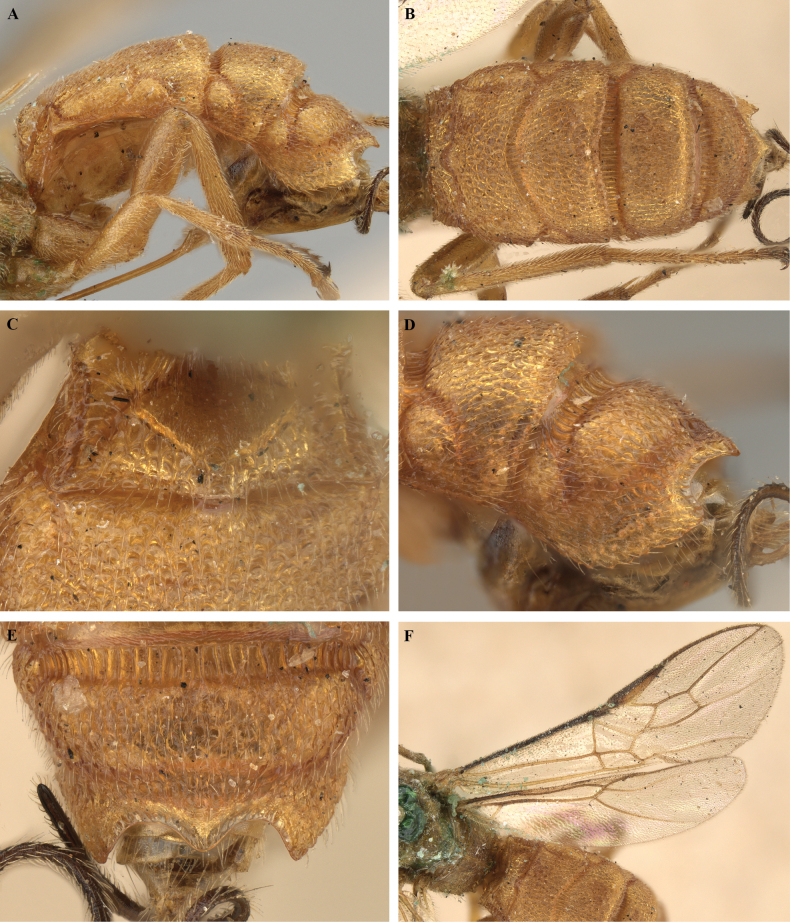
*Trigastrothecatrilobata* ♀ holotype **A** metasoma, lateral view **B** metasoma, dorsal view **C** T1, dorsal view **D** TT4 and 5, lateral view **E** T5, dorsal view **F** wings.

## ﻿Discussion

There were previously seven valid described species of *Trigastrotheca* described from mainland Africa (including two based on males described under the generic name *Kenema* but excluding *M.inermis*), six from Asia, and one from Australia (Raweearamwong et al. 2020). The additional species described here extend its known distribution to Madagascar, and collectively result from the work of individual collectors and more recently by large Malaise trapping initiatives (e.g. Quicke et al. 2023b).

The molecular phylogenetic tree presented (Fig. 1) lacks strong support for most of the deeper nodes but some small groups of species are well supported. The sequenced species from Thailand comprise two separate clades, one containing three species that share the very similar (black, yellow, and white) color patterns to *T.tridentata*; however, the latter is recovered separately being strongly (99% bootstrap) as sister group to *T.sureeratae* Quicke & Butcher, 2017, which has a rather different, predominantly yellow brown pattern, though still with a black and white metasoma. Unfortunately, *T.sureeratae* is known only from males so it is not currently possible to compare potentially more informative characters of the posterior metasomal tergite.

As regards the Afrotropical species known only from males and have virtually entirely ochreous yellow bodies (van Achterberg and Sigwalt 1987), we consider that there is insufficient material and, in particular, species for which members of both sexes are known, that it would be imprudent to attempt to incorporate them into the identification key with females. We believe that it will only be possible to integrate the species based only on a single male holotype into the study when fresh material (with correct identification) and ideally also molecular data, are available. In the case of other species, we have found that qualitative differences in color pattern adequate for correct identification in most cases. Regarding *Trigastrothecaquickei* van Achterberg, 1983, from Sierra Leone, the original description does not mention the color of the stemmaticum, but there is no indication that it is darker that the rest of the head from the figure, in which case it would run in our key to *T.carinata* sp. nov. which comes from India. *Trigastrothecaserrata* van Achterberg & Sigwalt, 1987, from Senegal, would run to couplet 4, but cannot be taken further since the serration of the T5 is probably not comparable between males and females.

## Supplementary Material

XML Treatment for Trigastrotheca aethiopica

XML Treatment for Trigastrotheca braeti

XML Treatment for Trigastrotheca carinata

XML Treatment for Trigastrotheca christianhenrichi

XML Treatment for Trigastrotheca flava

XML Treatment for Trigastrotheca formosa

XML Treatment for Trigastrotheca freidbergi

XML Treatment for Trigastrotheca griffini

XML Treatment for Trigastrotheca khaoyaiensis

XML Treatment for Trigastrotheca naniensis

XML Treatment for Trigastrotheca simba

XML Treatment for Trigastrotheca similidentatata

XML Treatment for Trigastrotheca sublobata

XML Treatment for Trigastrotheca acroceropsis

XML Treatment for Trigastrotheca nigricornis

XML Treatment for Trigastrotheca romani

XML Treatment for Trigastrotheca sureeratae

XML Treatment for Trigastrotheca tricolor

XML Treatment for Trigastrotheca tridentata

XML Treatment for Trigastrotheca trilobata

## ﻿Additional information

### Author ORCIDs

Donald L. J. Quicke https://orcid.org/0000-0003-4471-6775

Simon Van Noort https://orcid.org/0000-0001-6930-9741

Avunjikkattu Parambil Ranjith https://orcid.org/0000-0001-7061-9659

Ariel L. L. Friedman https://orcid.org/0000-0003-4389-6858

Hans Mejlon https://orcid.org/0000-0003-0815-4656

Buntika A. Butcher https://orcid.org/0000-0002-0541-0709

### Data availability

All of the data that support the findings of this study are available in the main text.
